# Sensitization strategy for sonodynamic therapy

**DOI:** 10.7150/thno.130498

**Published:** 2026-03-30

**Authors:** Sinong Li, Ziyi Yang, Ying Zhang, Genyuan Zhang, Zoujuan Cai, Yihan Xie, Zhiqun Bai, Jin Sun, Hongzan Sun, Zhiguang Chen, Liang Sang

**Affiliations:** 1 Department of Ultrasound, the First Hospital of China Medical University. China Medical University, No.155, Nanjing North Road, Shenyang 110001, China.; 2 Department of Pharmaceutics, Wuya College of Innovation, Shenyang Pharmaceutical University, Shenyang, 110016, China.; 3 Joint International Research Laboratory of Intelligent Drug Delivery Systems, Ministry of Education, Shenyang, 110016, China.; 4 Department of Nuclear Medicine, Shengjing Hospital of China Medical University. Shenyang, Sanhao Street No.36,110004, China.

**Keywords:** sonodynamic therapy, piezoelectric sonodynamic therapy, sonosensitizers, sonodynamic immunotherapy

## Abstract

Sonodynamic therapy (SDT), a tumor treatment modality characterized by deep tissue penetration and high spatiotemporal precision, faces multiple challenges in clinical translation, including suboptimal sonosensitizer efficiency, suppression by the tumor microenvironment (TME), and insufficient induction of antitumor immune responses. This paper systematically reviews the multifaceted sensitization strategies of SDT, breaking away from traditional single-optimization approaches to establish a comprehensive synergistic enhancement framework spanning the entire chain involving “material design-microenvironment regulation-immune remodeling-synergistic therapy.” In sonosensitizer engineering, current research emphasizes advanced design strategies—including defect engineering, heterostructure construction, and piezoelectric materials—to markedly enhance reactive oxygen species (ROS) generation through band structure modulation and mechano-electro-chemical coupling effects. To overcome the TME-associated constraints, a series of innovative strategies such as hypoxia alleviation, antioxidant depletion, metabolic reprogramming and bacteria-mediated targeted delivery have been developed to mitigate ROS scavenging and improve tumor selectivity. Furthermore, this review summarizes how SDT is integrated with multiple synergistic modalities such as chemodynamic therapy, phototherapy, immunotherapy, and ferroptosis/cuproptosis induction and systematically elucidates the underlying mechanisms and therapeutic potential of these combinations in triggering immunogenic cell death, reversing tumor immunosuppression, and ultimately enabling diagnostic-therapeutic integration. Despite persistent challenges in nanodelivery, controllable ROS generation, and clinical standardization, this review highlights that the development of multimodal, responsive, and biohybrid platforms is driving SDT toward a new paradigm of precise and intelligent cancer treatment. Collectively, these findings provide a systematic strategic blueprint with translational potential for treating deep-seated and drug-resistant solid tumors.

## 1. Introduction

The concept of sonodynamic therapy (SDT) dates back to the late 1980s and early 1990s, emerging directly from efforts to overcome the limited tissue penetration inherent to photodynamic therapy (PDT). In 1989, Umemura *et al.* 1 reported that hematoporphyrin markedly enhanced ultrasound-induced cytotoxicity in Ehrlich ascites carcinoma cells, and the term “sonodynamic therapy” was formally proposed in 1992. Currently, early-stage solid tumors are primarily treated by surgical resection 2, whereas advanced or metastatic tumors are generally managed with chemotherapy, radiotherapy, or immunotherapy 3. Although chemotherapy has demonstrated therapeutic efficacy in certain tumor types 4, its clinical application is limited by long-term toxicity resulting from nonspecific cytotoxic effects 5. Immunotherapy has achieved substantial success in hematologic malignancies; however, response rates in solid tumors remain limited 6, with most patients experiencing primary or acquired resistance 7. Moreover, the hypoxic tumor microenvironment markedly reduces radiotherapy sensitivity; even with fractionated irradiation strategies, hypoxia-associated resistance remains a major therapeutic challenge 8. Owing to its noninvasive characteristics, high targeting precision, and ability to modulate the tumor microenvironment, SDT has emerged as a promising strategy to address these therapeutic limitations. However, because of the intrinsic limitations of conventional organic sonosensitizers—poor aqueous solubility, limited tumor-targeting capability, and inadequate physicochemical stability 9, 10—together with an incompletely understood mechanism of action, SDT has been largely restricted to preclinical laboratory investigations, with overall therapeutic efficacy remaining unsatisfactory. The advent of nanotechnology has emerged as a transformative driving force accelerating the rapid development of SDT. Researchers 11, 12 have developed multifunctional nanoplatforms that serve either as carriers for sonosensitizers or as intrinsic sonosensitizers, enabling tumor-targeted delivery and spatiotemporally controlled release of therapeutic agents. More importantly, a range of novel inorganic nanoscale sonosensitizers—such as titanium dioxide, black phosphorus, molybdenum disulfide and carbon dots (CDs)—as well as organic nanoassemblies have been developed 13. By means of bandgap engineering, heterojunction construction, and defect modulation, these materials markedly increase ROS generation efficiency and quantum yield 14, 15. Currently, SDT research has entered a stage of intelligent and synergistic integration, focusing on the development of integrated diagnostic-therapeutic systems that combine diagnostic imaging, TME regulation, and multimodal therapeutic strategies 16. The core challenge centers on “sensitization strategies,” which seek to overcome the intrinsic limitations of SDT through coordinated material engineering, microenvironment modulation, and synergistic therapeutic interventions.

However, SDT still faces numerous challenges and shortcomings in advancing toward mature clinical translation. First, the lack of ideal sonosensitizers is the primary bottleneck. Although nanomaterials hold great promise, issues such as their long-term biosafety, metabolic pathways *in vivo*, potential immunogenicity, and large-scale standardized production require urgent resolution. Second, the tumor microenvironment imposes significant constraints. Hypoxia 17 and the overexpression of antioxidants (such as glutathione) 18, which are prevalent within solid tumors, severely deplete ROS and diminish therapeutic efficacy. Finally, the lack of standardization in treatment systems and parameters—including the optimal combination of ultrasound frequency, intensity, irradiation duration, sonosensitizer dosage and administration window—has yet to be established, thereby affecting treatment reproducibility and efficacy assessment. In recent years, numerous synergistic strategies have been developed to overcome these limitations. For instance, integrating SDT with chemotherapy 19, chemodynamic therapy (CDT) 20, PDT, and/or photothermal therapy (PTT) 21 can improve tumor targeting; increase ROS generation, and alleviate tumor hypoxia, excessive antioxidant capacity, and immunosuppression within the TME, thereby maximizing therapeutic efficacy. This review systematically summarizes the following sensitization strategies in SDT: (i) increasing the efficiency of ROS generation, (ii) improving tumor selectivity, (iii) regulating the TME, and (iv) enhancing the effects of cytotoxicity, beginning with the molecular mechanisms and physicochemical principles underlying SDT and then moving on to an in-depth analysis of the advantages and limitations of diverse combination approaches and their potential for clinical translation. Overall, this work offers a theoretical framework and forward-looking perspective to advance the application of SDT in the precise treatment of solid tumors.

## 2. SDT Mechanism of Operation

SDT is a tumor treatment modality that relies on the synergistic interaction of ultrasound, sonosensitizers, and molecular oxygen. Compared with HIFU, SDT generally utilizes low-intensity focused ultrasound (LIFU, Power 0.5-3.0 W/cm^2^, Frequency 1.0-2.0 MHz), which minimizes skin damage, enables deeper tissue penetration, and ensures superior safety 22. SDT activation is mediated by the interplay of acoustic energy input, mechanical effects, and localized thermal effects. Notably, under clinically relevant ultrasound conditions, mechanical effects are widely considered to play a dominant role. Its core principle lies in harnessing the physical effects generated by ultrasound within biological tissues to stimulate sonosensitizers, triggering a cascade of photonic, electrical, chemical, and mechanical responses. Ultimately, this induces tumor cell apoptosis or necrosis by generating highly cytotoxic ROS or directly disrupting cellular structures 23, 24. During propagation through liquid media, ultrasound generates periodic pressure oscillations that induce the formation and dynamic evolution of microbubbles 25, resulting in two distinct cavitation phenomena: stable cavitation and inertial cavitation. Stable cavitation involves the oscillatory expansion and contraction of microbubbles under ultrasonic irradiation, producing localized microstreaming, shear stress, and acoustic microjets. Although it does not directly generate free radicals, these mechanical effects reduce the energy threshold for cell membrane disruption in the presence of sonosensitizers, thereby facilitating localized tumor cell apoptosis. Inertial cavitation represents a more violent cavitation regime, in which microbubbles undergo rapid growth followed by catastrophic collapse after they accumulate ultrasonic energy, generating transient extreme temperatures and pressures. These conditions can directly disrupt cell membranes and cytoskeletal integrity, increase membrane permeability, and simultaneously induce acoustically driven phenomena such as sonoluminescence and localized acoustic heating, thereby further enhancing overall ROS generation efficiency 26, 27. The predominant ROS species generated vary depending on the physicochemical properties of the sonosensitizer and the local microenvironment, including singlet oxygen, hydroxyl radicals, and superoxide anions.

**Figure 1:**
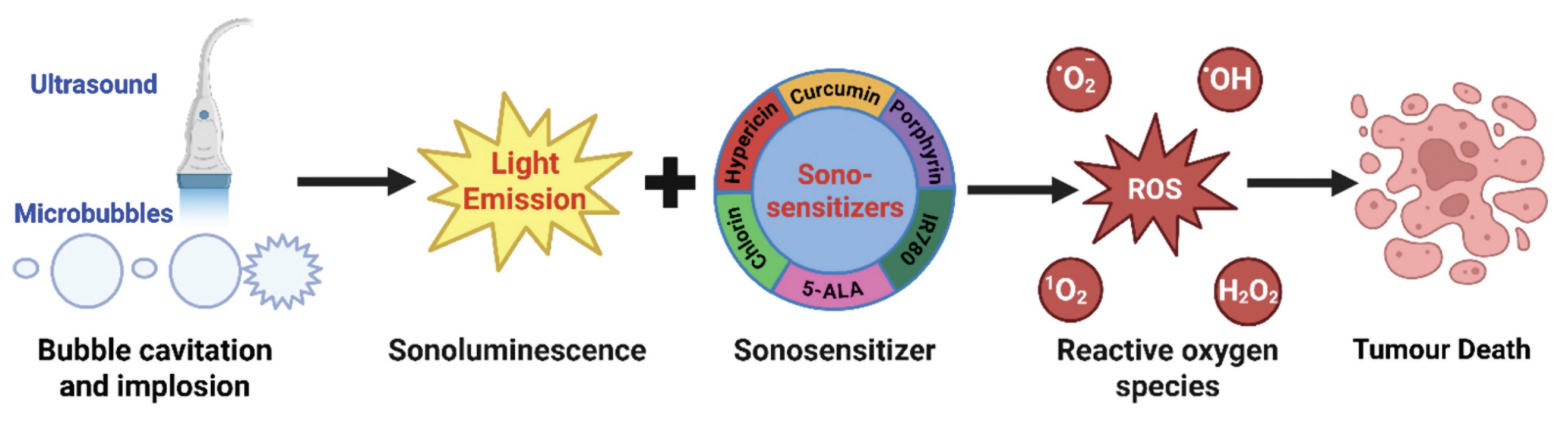
SDT mechanism of operation (Microbubbles undergo cavitation and rupture under ultrasonic stimulation, thereby generating sonoluminescence; sonoluminescence activates sonosensitizers, such as IR780, 5-ALA, etc., inducing electron-hole pair separation to produce reactive oxygen species, leading to cell death.). Adapted with permission from 28, *copyright 2025 The Authors. Published by Elsevier B.V*.

Overall, the mechanisms of SDT can be broadly classified into two principal pathways: (i) ROS-independent mechanical cellular damage, arising from the synergistic effects of stable cavitation and sonosensitizers; and (ii) ROS-dependent chemical biological damage, mediated by the combined action of inertial cavitation and sonosensitizer-catalyzed reactions. This therapeutic modality offers distinct advantages, including deep tissue penetration, high spatiotemporal precision, and excellent compatibility with combination strategies such as chemotherapy and immunotherapy. Notably, it shows substantial promise for effective deep-tumor targeting when integrated with nanomedicine-based delivery systems. Therefore, this review systematically summarizes and provides perspectives on sensitization strategies targeting the core components of SDT, namely sonosensitizers and oxygen availability (Figure 1).

## 3. SDT Sensitization Strategy - Improvements to Sonosensitizers

**Figure 2:**
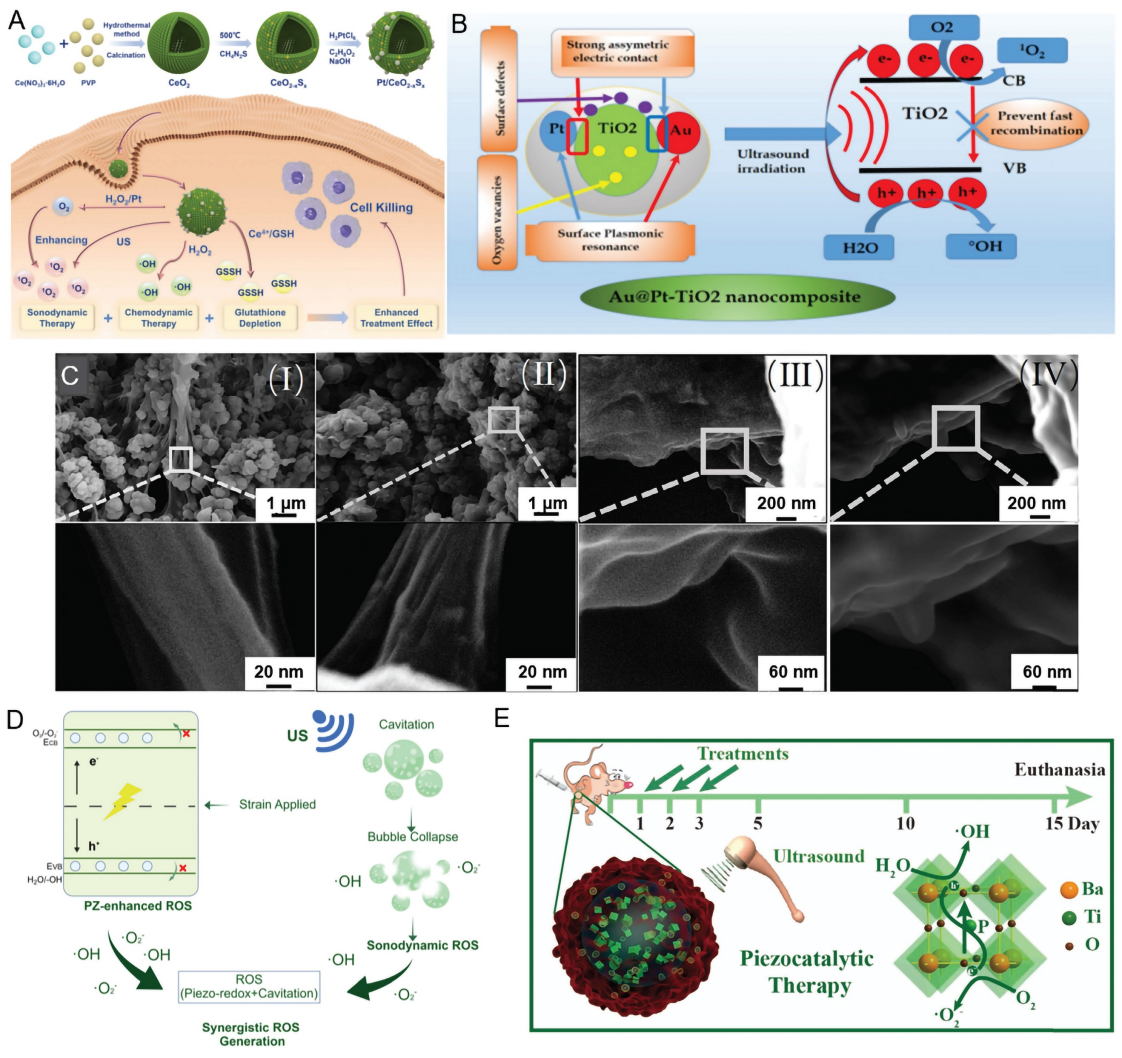
Improvement strategies for sonosensitizers. A) Pt/CeO2xSx schematic diagram of the preparation process and therapeutic mechanism of nanoparticles. Adapted with permission from 29, *copyright Royal Society of Chemistry 2024* B) A proposed mechanism for the contribution of the Au@Pt-TiO_2_-nano-GO components in the sonosensitizing enhancement. Adapted with permission from 39, *copyright The Author(s), under exclusive licence to Springer Science+Business Media, LLC, part of Springer Nature 2025;* C) SEM image of rP(VDF-TrFE) and P(VDF-TrFE) nanoparticles: (I) SEM of rP(VDF-TrFE) nanoparticles; (II) SEM of P(VDF-TrFE) nanoparticles; (III) Amorphous region of P(VDF-TrFE) nanoparticles, before recrystallization; (IV) Amorphous region of P(VDF-TrFE) nanoparticles, after recrystallization. Adapted with permission from 37, *copyright 2023 American Chemical Society;* D) Schematic diagram of piezoelectric effect, cavitation and ROS, *Created with BioGDP.com* 40; E) Schematic illustration of piezocatalytic therapy *in vivo*. Adapted with permission from 41, *copyright 2020 WILEY-VCH Verlag GmbH & Co. KGaA, Weinheim*.

The optimization of sonosensitizers constitutes one of the most fundamental strategies for enhancing SDT efficacy. Research has progressed from the direct repurposing of photosensitizers toward advanced materials engineering approaches that rationally design and regulate the electronic structure, band structure properties, and microstructural features of sonosensitizers, thereby maximizing ROS generation under ultrasonic stimulation. Specifically, defect engineering and heterojunction construction involve modulating the rapid electron-hole recombination in conventional sonosensitizers, thereby increasing the efficiency of ROS generation. For example, Zhang *et al.* 29 constructed a Pt/CeO₂-ₓSₓ Schottky heterojunction by introducing sulfur doping and oxygen vacancies into CeO₂, followed by the *in situ* deposition of Pt nanoparticles. This structure effectively promotes the separation of ultrasonically excited charge carriers, thereby significantly enhancing ROS generation (Figure 2A). Geng *et al.* 30 developed a degradable Co₉S₈₋ₓ nanocage sonosensitizer based on sulfur vacancy engineering. By precisely modulating the sulfur vacancy concentration, the bandgap was narrowed from 2.06 eV to 1.54 eV, which led to 2.6-fold and 9.6-fold increases in the ultrasound-triggered generation efficiencies of ¹O₂ and •OH, respectively.

Similarly, in classical TiO₂-based sonosensitizer systems, the incorporation of noble metals (e.g., Au and Pt) to construct heterostructures (such as Au@TiO₂-rGO and Au@Pt-TiO₂) effectively suppresses electron-hole pair recombination via interfacial electric field effects (Figure 2B). Moreover, these modifications endow the materials with additional enzyme-like catalytic activities (such as peroxidase- and glucose oxidase-like functions), thereby enhancing ROS generation through multiple synergistic pathways 31, 32. CDs have emerged as promising materials for tumor photodynamic therapy owing to their favorable optical properties, ultrasmall size, low toxicity, and excellent biocompatibility. Metal doping not only imparts ultrasonic responsiveness to CDs, enabling their application as potential sonosensitizers, but also modulates their bandgap structure, thereby facilitating rapid electron-hole separation under ultrasonic excitation and enhancing ROS generation 33. Liu *et al.* 34 developed folic acid-functionalized manganese-doped carbon dots (FA-Mn-CDs) as multifunctional theranostic agents for fluorescence/magnetic resonance (FL/MR) dual-modality imaging-guided SDT combined with CDT. In addition, Geng *et al.* 35 exploited the narrow bandgap and long-lived triplet excited states of near-infrared phosphorescent carbon dots to construct a near-infrared imaging-guided SDT platform, providing a new platform for precision tumor therapy. In addition, the development of novel sonosensitizers with piezoelectric properties has emerged as a promising frontier in SDT. Our team previously proposed the concept of sono-piezoelectric dynamic therapy (SPDT) 36 and developed recrystallized P(VDF -TrFE) -based composite piezoelectric nanoparticles (rPGd NPs@RGD) (Figure 2C) for targeted therapy and MRI-guided imaging of brain gliomas. We found that heat treatment optimizes the microstructure of disordered convoluted regions within the nanoparticles, thereby increasing their polarity, and overall piezoelectric performance. Under ultrasonic excitation, these nanoparticles efficiently generate ROS, thereby markedly inhibiting tumor cell proliferation 36, invasion, and migration. It is proposed that the randomly convoluted (amorphous) regions within P (VDF -TrFE) nanoparticles constitute the core of SPDT 37. Moreover, ultrasound-responsive piezoelectric electrical stimulation has been shown to significantly increase the efficacy of SDT against HER2-positive breast cancer cells 38.

Piezoelectric materials, as an emerging class of sonosensitizers, are characterized by their ability to directly convert ultrasonic mechanical energy into electrical energy via the piezoelectric effect, thereby efficiently driving sonodynamic therapeutic processes. Unlike conventional sonosensitizers, which predominantly rely on cavitation- or sonoluminescence-mediated processes to indirectly generate reactive oxygen species, piezoelectric sonosensitizers undergo lattice deformation under ultrasonic stimulation. This deformation induces polarization by separating positive and negative charge centers, thereby generating an intrinsic electric field that promotes efficient electron-hole pair separation and directional migration toward the material surface. When the electron potential of the conduction band is more negative than the O₂/O₂·⁻ redox potential (-0.33 V), molecular oxygen can be reduced to superoxide anions. Conversely, if the valence band potential exceeds the H₂O/•OH redox potential (+2.01 V), the holes are capable of oxidizing water to generate hydroxyl radicals. This enables highly efficient and controllable ROS generation 41 (Figure 2D). Additionally, certain piezoelectric materials (such as barium titanate) can generate piezoelectric potentials reaching 2.9 V, which are sufficient to induce depolarization of mitochondrial and cytoplasmic membranes. This directly disrupts the cellular electrophysiology and induces apoptosis, establishing a synergistic therapeutic mechanism that operates through combined mechanical, electrical, and chemical modalities. This class of materials comprises diverse systems, including inorganic piezoelectric ceramics (such as ZnO and BaTiO₃), organic piezoelectric polymers (such as PVDF-TrFE), and natural piezoelectric biomaterials (such as glycine crystals). Each material has distinct advantages in terms of biocompatibility, degradability, and functional tunability. In recent years, research on piezoelectric materials has shifted its focus toward optimizing performance through material modification. These include strategies such as metal doping (such as Mn -ZnO), constructing heterojunctions (such as Cu₂₋ₓO-BaTiO₃), surface functionalization (such as antibody-modified BaTiO₃), and developing composite piezoelectric materials (such as PVDF -TrFE-based nanocomposites). These approaches collectively aim to suppress electron-hole pair recombination, increase piezoelectric coefficients, improve tumor-targeting efficiency and biocompatibility, and enable synergistic integration with chemotherapy and immunotherapy. Notably, these advances have also given rise to emerging concepts such as piezoelectric-catalyzed immunotherapy 42. Upon ultrasound stimulation, piezoelectric materials generate reactive oxygen species (ROS) via electron-hole separation and subsequently remodel the tumor immune microenvironment through several interconnected mechanisms: inducing immunogenic cell death (ICD) through activation of the cGAS-STING pathway, thereby alleviating the immunosuppressive activity of regulatory T cells (Tregs) and natural killer (NK) cells; promoting the polarization of M2 macrophages toward the proinflammatory M1 phenotype via the CAMK2A-NF-κB signaling pathway; and facilitating the metabolic conversion of lactate to pyruvate. Collectively, these synergistic effects markedly increase the intratumoral infiltration of M1 macrophages and CD4⁺ and CD8⁺ T cells, while concurrently reducing immunosuppressive Tregs and lactate accumulation, thereby eliciting effective antitumor immune activation 42.

Nevertheless, several critical challenges remain. Inorganic piezoelectric materials (such as BaTiO₃) exhibit excellent piezoelectric properties but often suffer from limited biocompatibility; organic polymers (such as PVDF -TrFE) exhibit excellent biocompatibility but relatively weak piezoelectric responses; and natural piezoelectric materials (such as β-glycine), despite their outstanding piezoelectric properties, are constrained by phase instability and susceptibility to polymorphic transitions under physiological conditions (Table 1). Future research should prioritize achieving an optimal balance between enhanced piezoelectric performance and biocompatibility while promoting the clinical translation of piezoelectric sonodynamic therapy through rational multiscale material design. Although the majority of SDT sonosensitizers remain at the preclinical stage, several clinically approved or clinically investigated agents, such as 5-aminolevulinic acid (5-ALA), hematoporphyrin monomethyl ether (HMME), and indocyanine green (ICG), have demonstrated sonodynamic activity, underscoring the translational potential of SDT 43, 44.

## 4. SDT Sensitization Strategy - Alleviating Tumor Hypoxic Microenvironments

**Figure 3:**
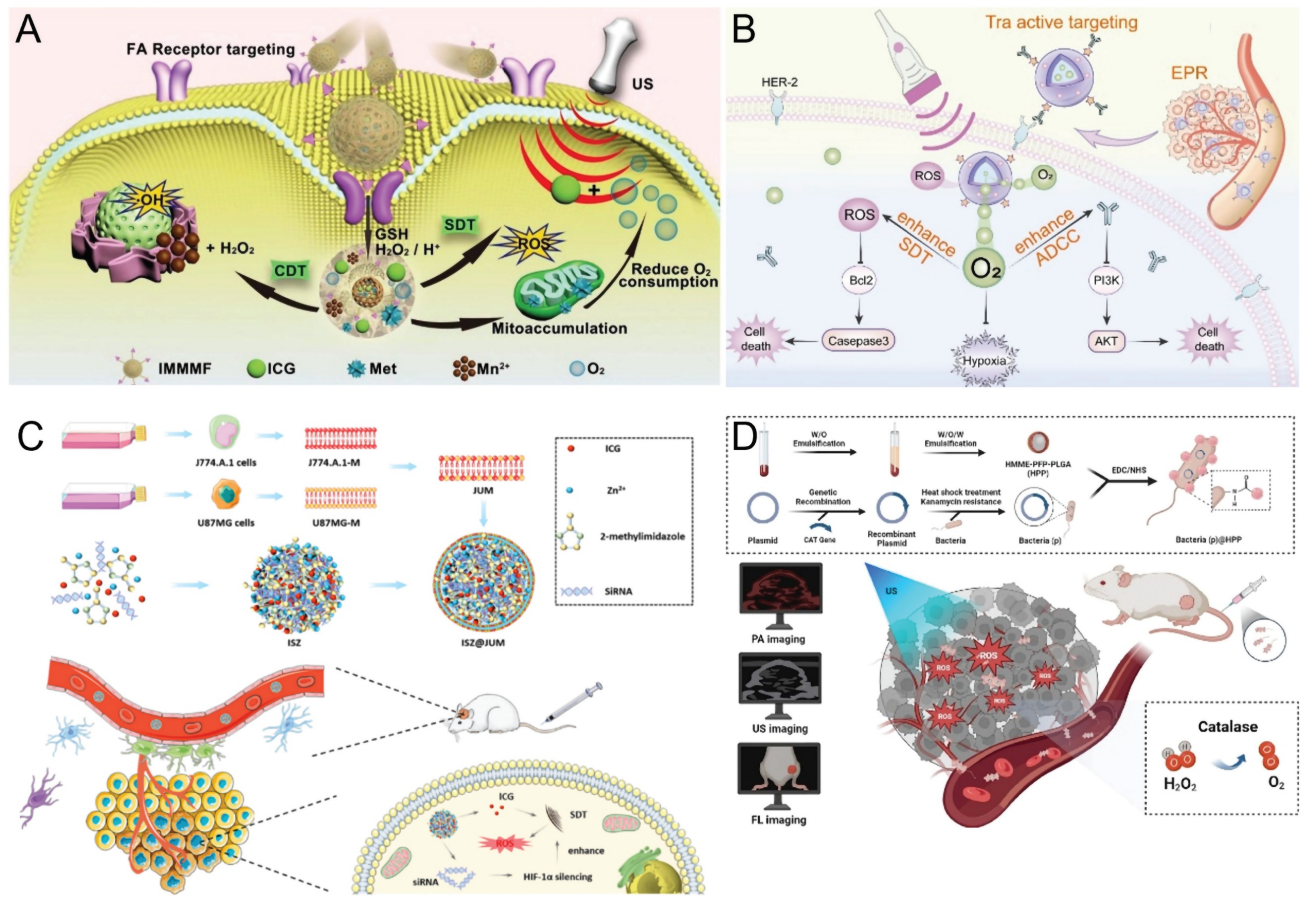
Alleviating tumor hypoxic microenvironments A) Synthesis of immunoglobulins and its application in CDT/SDT combination therapy. Adapted with permission from 49, *copyright 2024 Elsevier Inc.* B) Schematic illustration of the composition of TPPO NPs and the enhanced therapeutic effect achieved by combining trastuzumab with SDT in treating HER-2 positive gastric cancer through localized ultrasound irradiation triggering oxygen release. Adapted with permission from 52, *copyright 2025 Elsevier B.V.* C) Schematic diagram of ISZ@JUM preparation and enhanced SDT-mediated interference RNA release to suppress HIF-1αexpression. Adapted with permission from 56, *copyright 2023 American Chemical Society* D) Schematic of Bacteria (p)@HPP preparation and ROS generation upon ultrasonic irradiation to kill tumor cells while enabling PA/US/FL multimodal imaging. Adapted with permission from 59, *copyright 2024. Published by Elsevier Ltd*.

SDT predominantly relies on molecular oxygen to generate ROS; however, tumor hypoxia directly limits ROS production, thereby reducing its therapeutic efficacy 45. In addition, hypoxic conditions promote the stabilization of hypoxia-inducible factor-1α (HIF-1α) 46, which drives tumor metabolic reprogramming, angiogenesis, and mitochondrial fission, collectively enhancing cellular resistance to therapy. Previous studies 47 have shown that nanoparticles undergo a cascade reaction of H_2_O_2_ →O_2_ →·O_2_^-^ upon TME stimulation, thereby alleviating hypoxia during SDT. Manganese dioxide (MnO_2_) nanoparticles catalyze the conversion of endogenous H_2_O_2_ into tumor-infiltrating O_2_ while simultaneously depleting overexpressed GSH within tumors, providing a promising strategy to alleviate tumor hypoxia 48. Zhang *et al.* 49 developed hollow MnO₂ nanoparticles loaded with ICG and metformin to alleviate tumor hypoxia and enhance SDT. A tumor-targeted nanoplatform (IMMMF; Figure 3A) was further constructed by coating the nanoparticles with a folic acid-functionalized metal-phenolic network. In response to folate receptor overexpression 50, IMMMF preferentially accumulates in tumor tissues and is efficiently internalized. Within the tumor microenvironment, degradation of the nanoplatform releases Fe²⁺ and Mn²⁺ to induce CDT. Moreover, the released metformin suppresses mitochondrial complex I, reducing oxygen consumption and enhancing ICG-mediated SDT. In addition, MnO₂ catalyzes endogenous H₂O₂ to generate O₂ *in situ*. Together, reduced oxygen consumption and *in situ* oxygen generation further enhance SDT, enabling synergistic CDT/SDT.

Under hypoxic conditions, tumor cells adapt to oxygen deprivation through mechanisms such as upregulating HER-2 gene expression and HIF-1α protein expression and activating the PI3K/AKT signaling pathway 51, 52. HIF-1α is a key factor contributing to resistance to trastuzumab antibody-dependent cell-mediated cytotoxicity (ADCC), and its expression is directly regulated by HER-2 overexpression 53. Xie *et al.* 52 developed targeted ultrasound-sensitive nanoparticles (TPPO NPs) consisting of a perfluorooctyl bromide (PFOB) core, a Pyrrole Philippa liposome shell, and trastuzumab modification. By exploiting nanoparticle-mediated EPR effects and active targeting with trastuzumab, these NPs improve drug delivery efficiency. The oxygen self-supply capability of TPPO NPs alleviates tumor hypoxia, thereby enhancing the therapeutic efficacy of SDT and antibodies in treating HER-2 positive gastric cancer (Figure 3B). Moving beyond alleviating hypoxia, Wang *et al.* 54 exploited the hypoxic conditions of the TME by developing AIBA@MSN nanoparticles that generate oxygen-independent azo radicals, thereby disrupting the mitochondrial membrane potential and electron transport chain. This mitochondrial dysfunction induces tumor cell death and, when combined with PD-1 blockade, further enhances ICD.

SDT combined with gene therapy has been shown to alleviate the hypoxia of the TME and enhance the efficacy of tumor treatment. Gene therapy involves the artificial modification of cellular gene expression using nucleic acids. This approach involves identifying genes responsible for tumorigenesis and therapeutic resistance, designing exogenous nucleic acid sequences, introducing them into target cells, and using them to modulate specific molecular pathways to achieve antitumor effects. Therapeutic nucleic acids exist in several forms, including deoxyribonucleic acid (DNA), ribonucleic acid (RNA), and messenger RNA (mRNA), which promote the expression of tumor-suppressive genes; small interfering RNA (siRNA), short hairpin RNA (shRNA), and microRNA (miRNA), which silence oncogene expression; and immunostimulatory nucleic acids, which activate the host immune system to elicit stronger antitumor responses 55. While earlier studies focused primarily focused on improving the delivery efficiency of nanodrugs, current research emphasizes the development of advanced drug delivery systems capable of responding to the hypoxic, acidic, and redox-regulated TME. Li *et al.* 56 fused J774.A.1 macrophage membranes with U87 glioblastoma cell membranes to construct a hybrid biomembrane (JUM) possessing superior blood-brain barrier (BBB) permeability and glioblastoma-targeting, immune system camouflaging properties. This biomembrane was used to encapsulate the sonosensitizer ICG and HIF-1αsiRNA within acid-degradable ZIF-8 NPs, generating ICG-siRNA@ZIF-8 (ISZ) with high loading efficiency. The resulting ISZ@JUM (Figure 3C) nanoplatform exhibited remarkable BBB penetration and precise brain tumor-targeting ability. After it accumulates at the tumor site, the pH-responsive nanoplatform releases ICG and HIF-1α siRNA within tumor cells. Upon US irradiation, the released ICG produces large amounts of ROS, while the HIF-1α siRNA downregulates related genes to inhibit HIF-1α expression. This alleviates tumor hypoxia, thereby enhancing SDT efficacy under hypoxic conditions and achieving gene silencing-augmented SDT.

Additionally, bacterial therapy that leverages the hypoxic TME to alleviate hypoxia and enhance synergistic SDT efficacy has emerged as a promising therapeutic strategy. Bacterial cancer therapy, which employs live, attenuated, or inactivated bacteria and their components as therapeutic agents, has long been explored to directly suppress tumor growth or potentiate other anticancer treatments 57. Bacteria possess an intrinsic ability to migrate toward hypoxic regions and penetrate the complex TME 58, making them promising biological carriers for targeted cancer therapy. Wang *et al.* 59 developed an oxygen-generating, nanosonosensitizer-engineered bacterial biohybrid system (CAT-BL21@HMME-PFP-PLGA, CB@HPP), consisting of catalase-expressing engineered bacteria coated with a nanosonosensitizer, for multimodal imaging-guided SDT (Figure 3D). Through the tumor-targeting capability of engineered bacteria, nanosonosensitizers are selectively delivered to tumor sites, where catalase-mediated oxygen generation enhances the efficacy of SDT 60.

## 5. SDT Sensitization Strategy - Antioxidant Depletion

**Figure 4:**
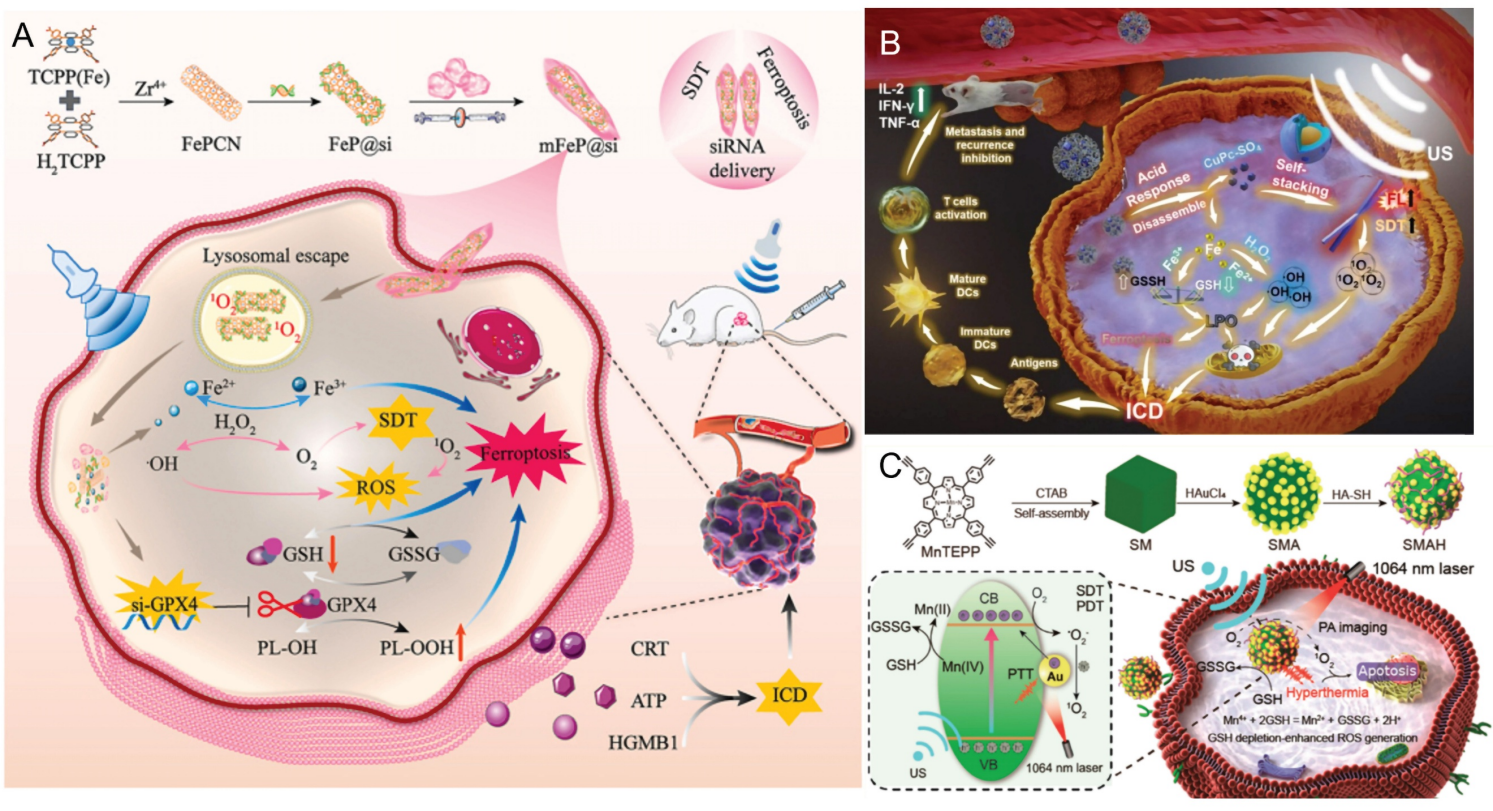
Antioxidant depletion. A) Schematic of mFeP@si preparation and its enhanced SDT under ultrasonic irradiation, coupled with interference RNA release to induce ferroptosis in cells. Adapted with permission from 64, *copyright 2024 The Authors.* B) Acid-sensitive CuPc-Fe@BSA nanocomposites synergize SDT/CDT and anticancer immune activation. Adapted with permission from 19, *copyright 2024 Elsevier Inc.* C) Preparation process of SMAH and the schematic diagram illustrating the integration of SMAH with SDT/PDT/PTT therapy. Adapted with permission from 65, *copyright 2023 American Chemical Society*.

The endogenous redox defense system of tumor cells dynamically maintains ROS homeostasis to limit their intracellular accumulation. GSH is the most abundant intracellular antioxidant, and it plays a critical role in maintaining redox homeostasis, facilitating drug detoxification, and protecting cells from oxidative damage induced by free radicals, peroxides, and toxins 61. However, its overexpression in cancer cells 18 protects them from excessive ROS-induced damage, thereby diminishing therapeutic efficacy. Multiple studies 62, 63 have designed nanoparticles to consume GSH, thereby preventing excessive depletion of ROS. To address the antioxidant nature of the TME, Sun *et al.* 64 developed a TME-responsive metal-organic framework (MOF)-based biomimetic nanosystem (mFeP@Si) for the delivery of siGPX4 and the implementation of SDT. The results demonstrated that the combined treatment with SDT and RNA interference (RNAi) precisely induced ferroptosis by increasing ROS production, depleting GSH, inactivating GPX4, and promoting the accumulation of toxic lipid peroxides, ultimately suppressing tumor progression (Figure 4A). The CuPc-Fe@BSA nanoparticles (10 nm) developed by Bai *et al.* 19 not only enhance the EPR effect and SDT efficacy but also release ferrous ions that exhibit glutathione oxidase (GSH-ox) and peroxidase (POD) activities, thereby promoting CDT and alleviating the highly antioxidant nature of the TME (Figure 4B). Xu *et al.* 65 constructed an SMA heterostructure that not only markedly enhanced ROS generation efficiency but also further promoted ROS accumulation by depleting intracellular GSH via the Mn^2+^/Mn^4+^ redox cycle. This strategy offers a valuable framework for enhancing the ^1^O_2_ yield from metal porphyrins and achieving synergistic therapeutic effects in SDT/PDT/PTT (Figure 4C). Wang *et al.* 66 employed TPA-OS, which functions as both a near-infrared (NIR) imaging agent and an organic sonosensitizer, to design and construct an intelligent sonosensitizer termed TPA-OS⊂CP5@CeOx.The acidic lysosomal environment triggers the release of TPA-OS, enabling its targeted translocation to mitochondria. Consequently, TPA-OS⊂CP5@CeOx has imaging-guided therapeutic effects at both the lysosomal and mitochondrial levels. By regulating the Ce³⁺/Ce⁴⁺ ratio and oxygen vacancy (V_O_) content, the system enhances the catalase (CAT)-like activity of CeOx and depletes GSH, thereby modulating the TME by alleviating hypoxia and attenuating the reducing conditions. These synergistic effects effectively enhance SDT performance, highlighting the potential of organic-inorganic hybrid sonosensitizers with integrated therapeutic and imaging functionalities.

## 6. SDT sensitization Strategy - Enhancing Reactive Oxygen Species Generation

### 6.1 SDT combined with CDT enhances ROS generation

**Figure 5:**
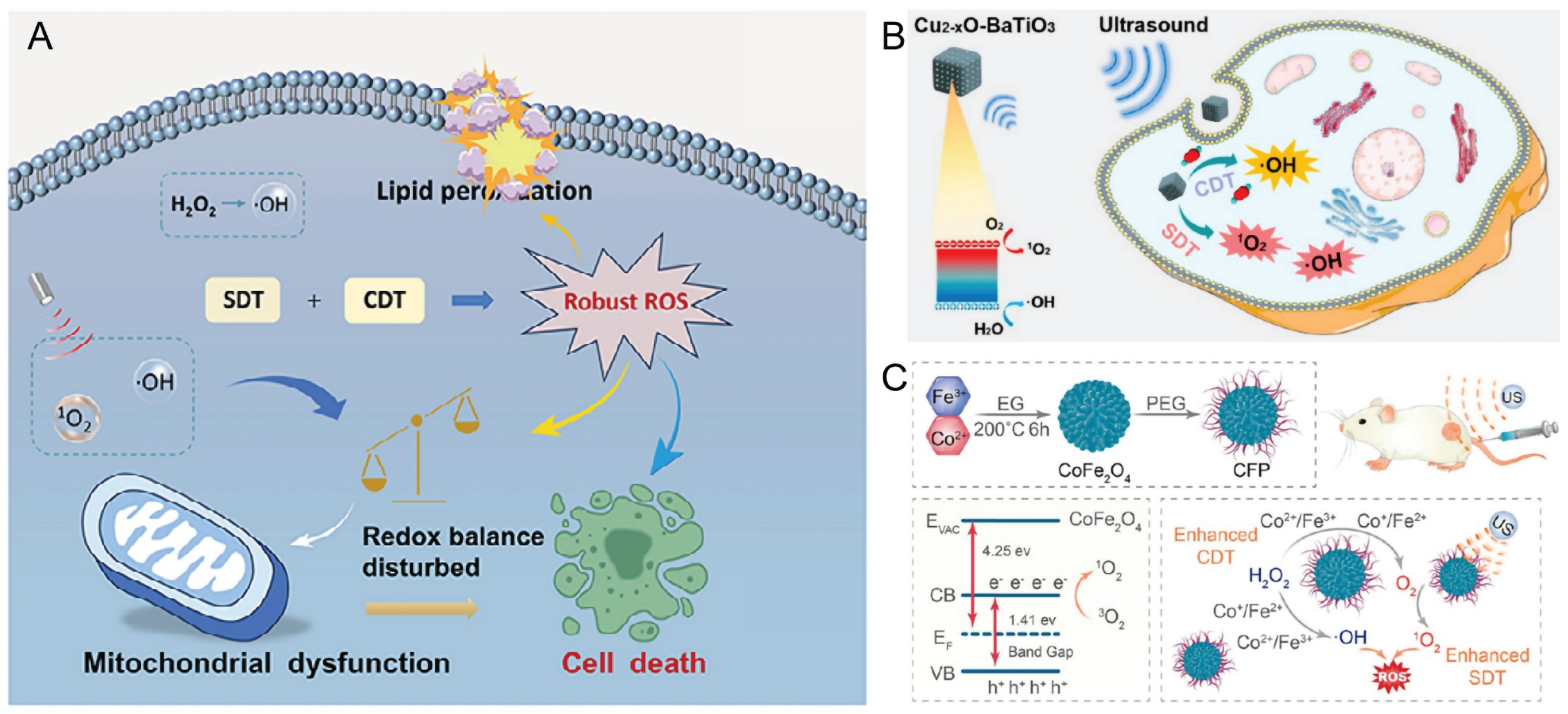
Enhancing reactive oxygen species generation—chemo-sonodynamic synergistic therapy A) Schematic illustration of the action of ErSbW for combined SDT and CDT to improve cancer therapy. Adapted with permission from 70, *copyright 2025 Wiley-VCH GmbH.* B) The process of Cu₂-ₓO-BaTiO₃ promoting the synergistic effect of SDT and CDT. Adapted with permission from 71, *copyright 2022 American Chemical Society.* C) Synthesis process of CFP and its mechanism of action in enhancing combined therapy of SDT and CDT. Adapted with permission from 72, *copyright 2021 American Chemical Society*.

CDT is an innovative tumor treatment strategy that exploits TME-specific chemical reactions, such as Fenton and Fenton-like reactions, to generate ROS *in situ*. The core mechanism involves the catalytic conversion of endogenous H_2_O_2_ into highly toxic •OH radicals through transition metal ions, thereby enabling the selective destruction of tumor cells 67. CDT depends on the overexpression of H_2_O_2_ and a mildly acidic TME. The ultrasonic cavitation effect decreases the mass diffusion resistance of substrates, thereby accelerating the rates of Fenton and Fenton-like reactions and enhancing •OH radical production from H_2_O_2_ under simulated TME conditions 68, 69. Chemo-sonodynamic synergistic therapy (CSDT) integrates the complementary advantages of CDT and SDT, making it particularly suitable for the treatment of deep-seated tumors. Du *et al.* 70 developed a novel sonosensitizing heterometallic oxoacid (ErSbW). This material features a precise capsule structure, excellent water solubility, high biocompatibility, and strong stability. The experimental results demonstrated that ErSbW efficiently catalyzes the generation of ROS for CDT. Upon ultrasonic irradiation, its catalytic efficiency increases threefold, indicating a pronounced synergistic enhancement. In melanoma models, ultralow doses of ErSbW achieved complete elimination of deep-seated tumors (Figure 5A). Zhao *et al.* 71 engineered Cu₂-ₓO-BaTiO₃ piezoelectric heterojunctions to overcome the limited efficiency of conventional inorganic sonosensitizers arising from rapid electron‒hole pair recombination. This material functions as both a sonosensitizer and a chemodynamic agent. Upon ultrasonic irradiation, the piezoelectric effect of the heterojunction efficiently promotes electron‒hole separation, markedly enhancing the generation of singlet oxygen (¹O₂) and hydroxyl radicals, thereby improving the efficacy of SDT. Concurrently, it catalyzes the conversion of tumor-derived endogenous H₂O₂ into •OH via a Fenton-like reaction, thereby enabling CDT (Figure 5B). Fu *et al.* 72 designed polyethylene glycol (PEG)-modified CoFe_2_O_4_ nanoflowers (CFPs) that effectively accumulate within tumor tissues. These CFPs catalyze the generation of •OH radicals via Fenton-like reactions for CDT and simultaneously react with endogenous H_2_O_2_ to produce O_2_. The elevated O_2_ concentration enhances SDT by promoting the formation of ^1^O_2_. The experimental results further confirmed that the CFP+H_2_O_2_+US group exhibited the highest ROS generation efficiency, thereby significantly improving the *in vivo* tumor-killing efficacy of SDT (Figure 5C).

In summary, because SDT relies on ultrasound excitation of sonosensitizers to generate ROS, and because CDT depends on H_2_O_2_ as a substrate to produce • OH radicals, the hypoxic and highly antioxidant TME substantially reduces the therapeutic efficacy of both modalities. Future research should therefore focus on alleviating tumor hypoxia and modulating the redox state within the TME to achieve sustained generation of ROS and H_2_O_2_. Moreover, LIFU may be insufficient to activate SDT in deep-seated tumors because of acoustic attenuation, whereas excessive ultrasound energy may damage surrounding healthy tissues. In addition to optimizing sonosensitizer performance, ultrasound parameters should be precisely tuned to ensure biological safety and minimize off-target effects.

### 6.2 SDT combined with PT enhances ROS generation

Phototherapy, primarily including PDT, PTT, and photoimmunotherapy (PIT), is a therapeutic modality that employs exogenous photoactive agents to increase the efficacy of light irradiation. Phototherapy achieves antitumor effects through the use of light to generate ROS or induce localized hyperthermia. PIT integrates the advantages of PT and immunotherapy to selectively eradicate cancer cells while eliciting polyclonal, tumor-specific immune responses 73.

**Figure 6:**
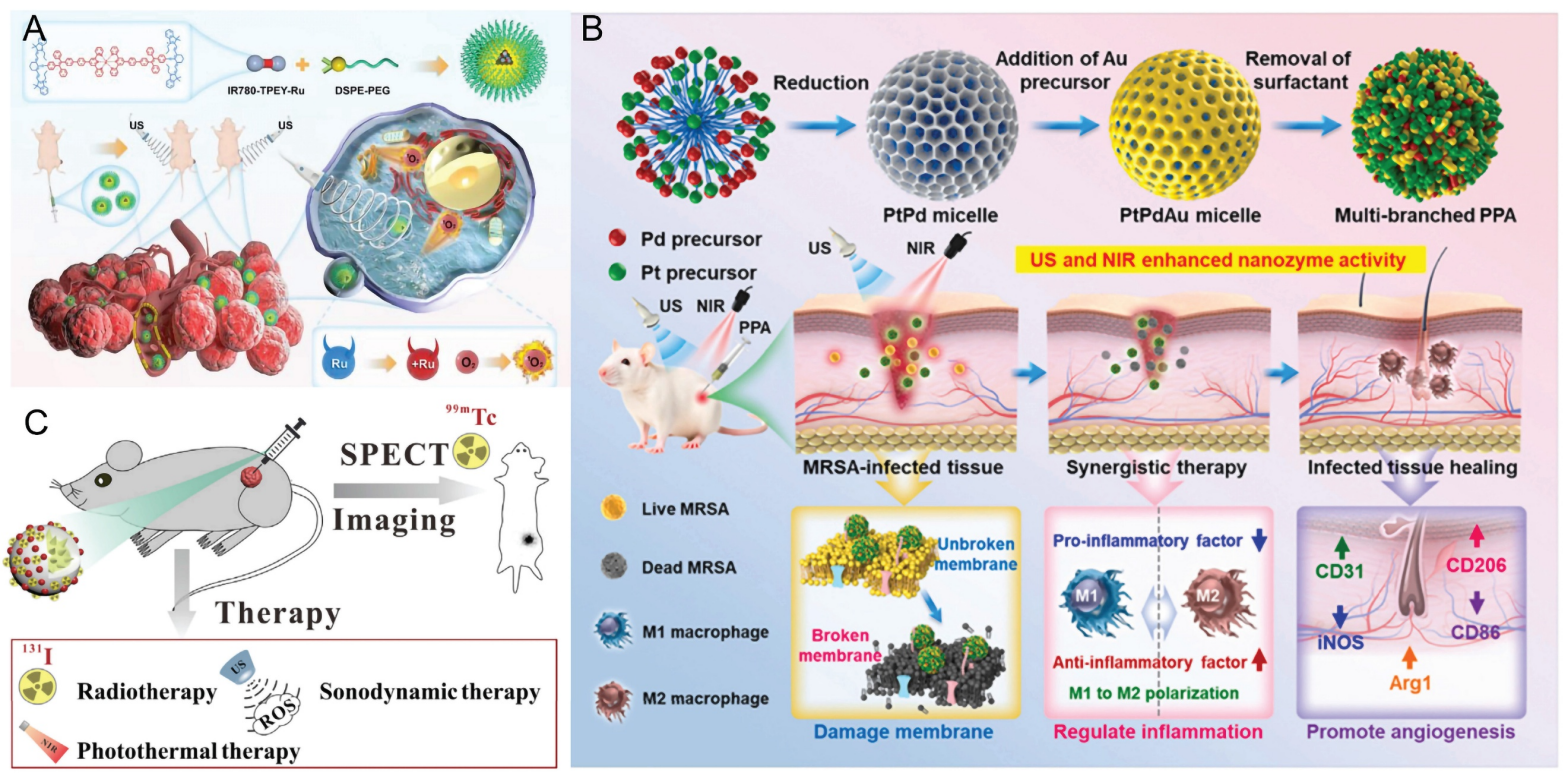
Enhancing reactive oxygen species generation—SDT combined with PT enhances ROS generation A) Synthesis routes of near-infrared ruthenium(II) Sonosensitizers and applications in SDT. Adapted with permission from 75, *copyright 2025 The Author(s). Published by Elsevier B.V.* B) US and NIR-amplified CDT for trimetallic alloy nanozymes to combat deep-seated MRSA. Adapted with permission from 21, *copyright 2025 The Authors.* C) Schematic illustration of ^131^I/^99m^Tc-AN@D/IX sonodynamic and photothermal combined therapy. Adapted with permission from 79, *copyright 2023 Elsevier Inc*.

Geng *et al.* 74 synthesized nanoscale amorphous manganese-protoporphyrin complexes (MnPPs) via carboxyl coordination between Mn^2+^ and the sonosensitizer protoporphyrin IX (PpIX). Upon light or US irradiation, MnPPs effectively converted O_2_ into ^1^O_2_. After 5 minutes of irradiation, 61.8% (light) and 32.4% (US) of 1,3-diphenylisobenzofuran (DPBF)—a classic ^1^O_2_ probe, was oxidized by MnPPs, confirming their high ROS-generation efficiency. Xu *et al.* 75 developed a ruthenium (II)-based sonosensitizer (IR780-TPEY-Ru) with excellent NIR absorption and emission properties. By integrating the π-expanded ligand IR780 with tetraphenylethylene, the resulting Ru(II) complex exhibited strong luminescence, high ^1^O_2_ generation efficiency, and excellent photostability 76, 77. This molecular design redshifts absorption into the biological optical window by modulating the frontier orbitals of the Ru center. Under ultrasound irradiation, IR780, IR780-TPEY, and IR780-TPEY-Ru all exhibit SDT activity, with IR780-TPEY-Ru showing the strongest therapeutic efficacy. These results indicate that synergistic coupling between the IR780 moiety and the Ru(II) center enhances ROS generation and antitumor efficacy (Figure 6A). He *et al.* 21 developed a platinum-palladium-gold ternary alloy nanozyme (PPA) as a synergistic platform to treat deep-tissue infections caused by drug-resistant bacteria. This platform integrates photothermal, chemodynamic, and sonodynamic therapies, while its hierarchical nanostructure facilitates biological membrane penetration. Upon dual activation by NIR laser and ultrasound, PPA generates heat and burst ROS production, achieving synergistic sterilization with a 95% inhibition rate. Multiomics analysis elucidates its multifaceted mechanisms, including modulation of oxidative stress, quorum sensing, and related pathways. Animal studies have shown that PPA effectively accelerates the healing of deep tissue infections while maintaining excellent biocompatibility, offering an integrated “diagnosis-treatment-regulation” strategy for managing deep-seated infections (Figure 6B). To increase ^1^O_2_ production, improve the efficiency of PDT and SDT, and leverage the ability of PTT to increase intratumoral blood flow 78, An *et al.* 79 developed the 131I/99mTc-AN@D/IX nanoparticles, which not only facilitate deep delivery but also generate ROS through SDT to further eliminate tumor cells, thereby enhancing the combined therapeutic effect of radionuclides and PTT (Figure 6C).

Owing to the extremely low penetration depth of visible light (< 3 mm) 80, PT has limited penetration depth through tissue. US can penetrate approximately 10 cm of deep tissue. However, sonosensitizers effective for both SDT and PT exhibit photosensitivity, necessitating strict light avoidance post-treatment until the sonosensitizer is metabolized to non-phototoxic levels. While the heat generated during PTT and the ROS accumulated in PDT/SDT effectively kill tumors, they may also cause collateral damage to surrounding healthy tissues or induce immune-related adverse reactions. Moreover, severe hypoxia within the TME significantly diminishes the therapeutic efficacy of PDT/SDT. Future research should focus on developing effective sonosensitizers for SDT/PT that can target tumor cells, alleviate the hypoxia in the TME, and mitigate biosafety concerns.

## 7. SDT Sensitization Strategy - Correcting Immunosuppression TME

### 7.1 SDT combined immunotherapy corrects immunosuppressive TME

Cancer immunotherapy activates the immune system to recognize and eradicate tumor cells by engaging both innate and adaptive immune responses 81, 82. Current immunotherapeutic strategies primarily include immune checkpoint blockade (ICB), adoptive T-cell therapy (ACT), therapeutic cancer vaccines, and cytokine- or antibody-based therapies 83. Despite their clinical promise, several major limitations persist, including low response rates, limited efficacy, and cytotoxicity resulting from insufficient immunogenicity. In 2018, Zhang *et al.* pioneered the application of SDT as an adjuvant immunotherapeutic strategy by utilizing sonosensitizers to enhance antitumor immunity 84. SDT not only directly induces tumor cell death but also converts nonimmunogenic cell death into ICD 54, 85, 86. ICD promotes the release or surface exposure of damage-associated molecular patterns (DAMPs), including high-mobility group box 1 (HMGB1), calreticulin (CRT), adenosine triphosphate (ATP), and heat shock proteins (HSPs). These molecules, along with tumor-associated antigens (TAAs) that are passively released into the TME from intracellular structures upon cell death, promote the maturation of dendritic cells (DCs) and enhance antigen presentation to cytotoxic T lymphocytes (CTLs) 87. Consequently, SDT increases T-cell infiltration, remodels the TME, and exerts dual antitumor effects through direct cytotoxicity and immune activation.

**Figure 7:**
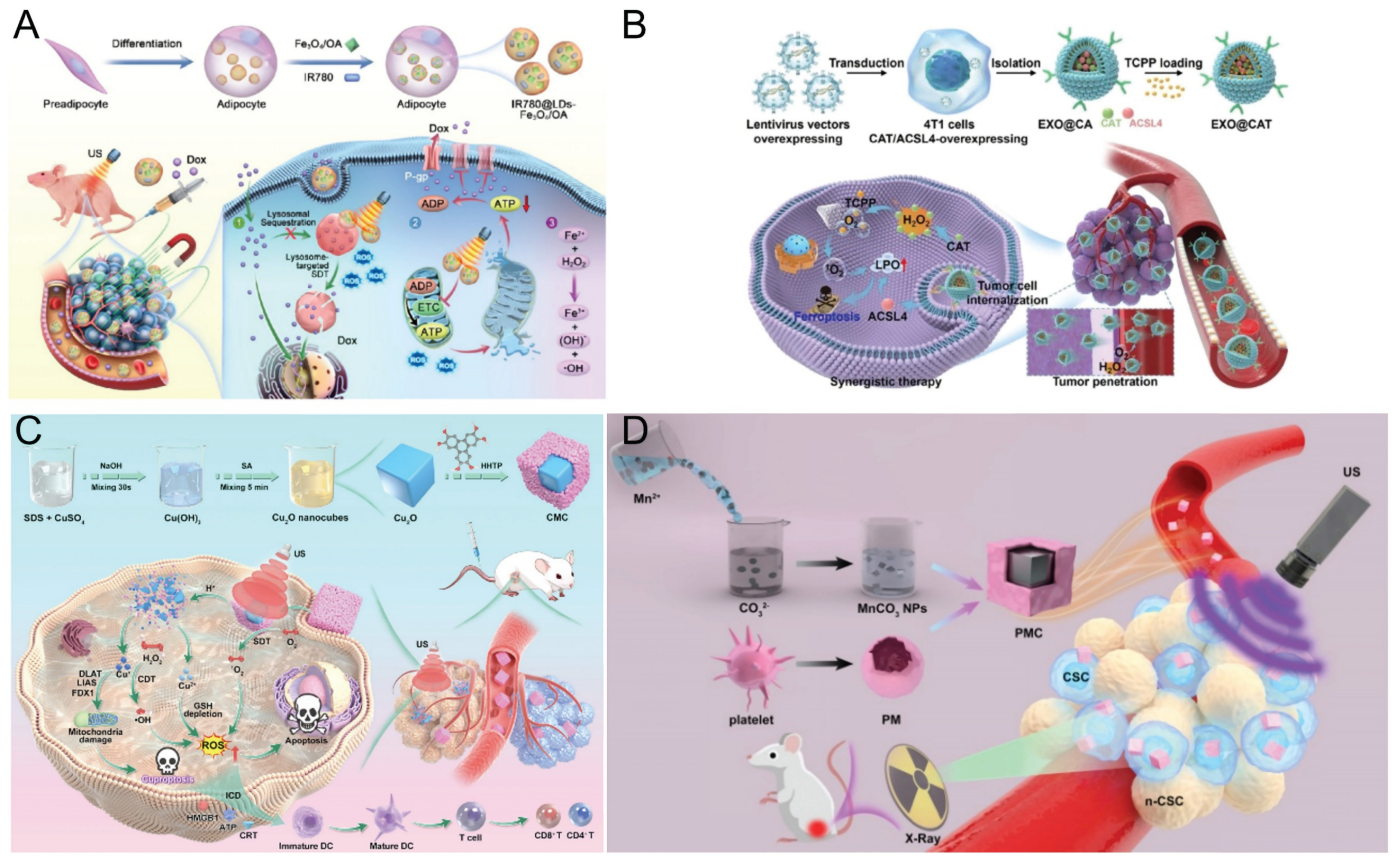
SDT sensitization strategy - correcting immune suppression in the TME A) Schematic diagram of RCM-Lip enhancing SDT activation of the cGAS-STING signaling pathway for synergistic immunotherapy. Adapted with permission from 88, *copyright 2025. Published by Elsevier Inc.* B) Schematic illustration of ML NPs achieving deep penetration and treatment of tumors. Adapted with permission from 100, *copyright 2025. Published by Elsevier Ltd.* C) Synthesis route of QD/POM1@NP@M and schematic diagram of enhanced SDT immunotherapy via inhibition of the CD39/CD73/ADO pathway. Adapted with permission from 113, *copyright 2025 The Authors.* D) Preparation of DOX@CWV and synergistic anticancer effects of DOX@CWV-mediated combined therapy of SDT, chemotherapy, and bacterial immunotherapy *in vivo*. Adapted with permission from 121, *copyright 2025 The Author(s). Advanced Science published by Wiley-VCH GmbH*.

To overcome the limitation that SDT-induced immune activation alone is often insufficient for complete tumor eradication, researchers 88-91 have developed nanoparticles incorporating stimulator of interferon genes (STING) agonists to activate the cyclic GMP-AMP synthase (cGAS)-STING signaling pathway and potentiate antitumor immunity. Yang *et al.* 88 developed a targeted liposomal formulation (RCM-Lip) co-encapsulating the sonosensitizer dihydroporphyrin e6 (Ce6) and the STING agonist MSA-2, modified with the cyclic arginine-glycine-aspartic acid (RGD) peptide on its surface to specifically bind integrin αvβ3, which is overexpressed on HCC cells (Figure 7A). This system was designed for ultrasound-assisted immunotherapy against HCC. The experimental results demonstrated that RCM-Lip-mediated SDT generated large amounts of ROS, induced tumor cell damage and elicited robust ICD, thereby effectively enhancing tumor immunogenicity. Moreover, activation of the STING pathway together with ICD promotes DCs maturation and CTLs infiltration into tumor tissues, stimulates the secretion of proinflammatory cytokines, and amplifies antitumor immune responses. The immunosuppressive characteristics of the TME not only limit antitumor immunotherapy but also compromise the efficacy of SDT because of features such as hypoxia, elevated GSH levels, and an acidic pH. Geng *et al.* 92 developed a single-atom catalyst, Pd_SA_/Ti_3-x_C_2_T_y_, capable of simultaneously modulating both immunosuppressive and SDT-inhibitory features of the TME. Specifically, compared with conventional Pd⁰ nanoparticles, Pd^δ+^ single-atom-doped nanoparticles exhibit superior electron-trapping capability and effectively suppresses electron-hole recombination, thereby markedly increasing the efficiency of ROS generation. Under US irradiation, the photogenerated holes in the valence band of Ti_3-x_C_2_T_y_ rapidly deplete overexpressed GSH in tumor tissues, thereby reducing ROS scavenging and further promoting ROS accumulation. In addition, the Pd^δ+^ single-atom catalyst exhibits catalase-like activity, effectively decomposing endogenous hydrogen peroxide and alleviating hypoxia within the TME. Ultimately, the elevated ROS levels generated under US stimulation not only induce ICD but also activate systemic antitumor immune responses, thereby remodeling the TME and enhancing the therapeutic efficacy of combined SDT-immunotherapy.

Although the combination of SDT and immunotherapy allows precise tumor targeting through sonosensitizers and produces abundant ROS under US irradiation to achieve selective tumor cell destruction and induce ICD, thereby amplifying antitumor immune responses, several critical challenges remain. These include the suboptimal biocompatibility of nanomaterials, the difficulty of sustaining stable ROS generation over time, and the limited targeting efficiency of immunotherapeutic agents. Moreover, tumor cells frequently upregulate immune checkpoint proteins such as programmed death-ligand 1 (PD-L1) in response to stress-induced inflammatory signaling, thereby establishing negative feedback loops that inhibit T-cell activation and facilitate immune evasion 93, 94. Notably, current combinations of SDT with immunotherapy mainly yield additive effects and have yet to elucidate or overcome the mechanistic interplay between the antioxidant suppression of ROS within the TME and tumor immune evasion. Moreover, careful attention should be given to the risks of excessive immune activation and the overproduction of ROS during treatment.

### 7.2 SDT combined GT activates immunosuppressive TME

Numerous studies 95-97 have demonstrated that nitric oxide (NO) not only acts as a crucial signaling molecule but also induces apoptosis of tumor cells. NO reacts with O_2_^-^ to form cytotoxic peroxynitrite (ONOO⁻) and modulates nitric oxide synthase (NOS) activity, thereby inhibiting angiogenesis and suppressing tumor progression. In addition, NO reduces extracellular matrix (ECM) stiffness, remodels its architecture, and enhances the permeability of both therapeutic agents and immune cells, ultimately improving treatment efficacy. Song *et al.* 98 conjugated the PD-L1 inhibitor BMS-1166 with a NO donor (Arg)9 via a thioketal linker to construct a ROS-responsive amphiphilic prodrug, BMS-1166-TK-(Arg)9. A piezoelectric nanodrug delivery system (BTO@BAL) was subsequently fabricated via self-assembly. This system consists of a barium titanate oxide (BTO) core decorated with the prodrug and the targeting peptide LFC131-PEG-DSPE. Under ultrasound irradiation, the piezoelectric BTO generates ROS 99, triggering TK cleavage and release of BMS-1166 and (Arg)9. The ROS subsequently induce NO release from (Arg)9, and released NO reacts with the ROS to form highly cytotoxic ONOO⁻, which disrupts the dense extracellular matrix, reducing tissue stiffness and enhancing drug penetration. Importantly, the synergistic ROS/NO interaction induces immunogenic cell death, while the ROS-triggered release of BMS-1166 downregulates PD-L1 expression, resulting in the remodeling of the immunosuppressive TME. Collectively, these mechanisms improve drug delivery efficiency and enhance immunotherapeutic efficacy, offering a promising multimodal strategy for pancreatic cancer.

The combination of SDT and GT has introduced novel strategies for modulating the immunosuppressive TME. However, owing to limitations in effective drug delivery and controlled release, Wang *et al.* 100 proposed a nanoparticle penetration strategy based on tumor pH gradients by exploiting the acidic TME. They constructed a self-electrophoresis-driven MgF₂@L-Arg nanoparticles (ML NPs; Figure 7B). MgF₂ nanoparticles were prepared by precipitation and subsequently loaded with L-Arg to obtain ML NPs. ML NPs generate an endogenous electric field driven by intratumoral proton gradients, enabling self-electrophoresis-mediated deep tumor penetration. MgF₂ NPs exhibit SDT activity and peroxidase-like catalysis to oxidize L-Arg for NO generation. Ultrasound-induced cavitation further enhances ROS generation, promoting synergistic SDT and GT. Moreover, the released Mg²⁺ enhances CD8⁺ T-cell cytotoxicity via LFA-1 engagement, amplifying antitumor immunity and suppressing metastasis 101.

Although the integrated therapeutic strategy combining SDT, GT, and immunotherapy holds great promise, several critical challenges remain to be addressed before successful clinical translation can be achieved. Second, the ICD induced by various gas molecules in combination with SDT demonstrates marked heterogeneity among different tumor types, highlighting the need for systematic screening to determine optimal therapeutic combinations for specific malignancies. Furthermore, owing to the unique characteristics of the TME, the synergistic strategy integrating of GT with SDT may be highly feasible only in specific cancer types, thereby increasing the complexity of optimizing precision oncology strategies.

### 7.3 SDT combined metabolic reprogramming corrects immunosuppressive TME

Metabolic reprogramming refers to the cellular process of altering metabolic pathways and energy production modes to adapt to environmental changes or sustain specific biological functions—a phenomenon particularly pronounced under pathological conditions. Various central metabolic pathways may be dysregulated in cancer cells 102. By competing for essential nutrients or impairing the metabolic flexibility of infiltrating immune cells, cancer cells can suppress antitumor immune responses 103, 104. Several studies 105-107 have demonstrated that altering the metabolic reprogramming of the TME synergizes with SDT to achieve immune activation. DAMPs released during ICD include ATP, which can stimulate T cell-mediated immunity by promoting macrophage polarization and enhancing DCs infiltration and antigen presentation 108, 109. Within the TME, extracellular ATP is rapidly converted into the immunosuppressive molecule adenosine (ADO) by the ectonucleotidases CD39 and CD73, which are often overexpressed in solid tumors 110. ADO increases cyclic adenosine monophosphate (cAMP) levels in T cells, thereby suppressing effector T cell proliferation and the secretion of proinflammatory cytokines 111. ADO can also drive the polarization of tumor-associated macrophages (TAMs) from an antitumor M1-like phenotype to a protumor M2-like phenotype. Additionally, ADO acts as a key effector molecule in Tregs, suppressing antitumor immunity by enhancing Treg activity 112. Through these mechanisms, ADO attenuates the immune-stimulating effects of ICD, thereby limiting the overall efficacy of cancer therapies and contributing to tumor recurrence. Zhang *et al.* 113 developed a nanocarrier consisting of a shell composed of poly (lactic-co-glycolic acid) (PLGA) and the cationic lipid 1,2-dioleoyl-3-trimethylammonium propane (DOTAP) modified with homologous tumor cell membranes, and a core containing the hydrophobic ultrasonic sensitizer Ag@S quantum dots and the hydrophilic CD39 inhibitor POM1. Upon administration, QD/POM1@NP@M efficiently accumulates at tumor sites because of the intrinsic homing ability of homologous tumor cell membranes (Figure 7C). Under ultrasound irradiation, Ag@S quantum dots produce high levels of ROS, inducing cell death and the sustained release of ATP. Concurrently, POM1 inhibits CD39-mediated ATP degradation in immune cells, resulting in elevated extracellular ATP levels within the TME. Elevated ATP enhances DCs maturation and antigen presentation, ultimately activating T cell-mediated immune responses. Additionally, the decrease in immunosuppressive ADO promotes macrophage polarization toward the M1 phenotype and suppresses Treg activity, thereby mitigating their immunosuppressive effects on T cells and contributing to comprehensive remodeling of immune cells and the TME.

Reprogramming of lipid metabolism is also recognized as a critical contributor to tumor drug resistance 114. Duan *et al.* 115 designed a metal-organic framework-based drug delivery system (OB@D-pMOF/CaP-AC, DDS) to simultaneously target the uptake of cholesterol esters (CE) and fatty acids, thereby modulating lipid metabolic reprogramming to overcome tumor drug resistance. MOFs efficiently generate ROS under ultrasonic irradiation and exhibit Fenton-like catalytic activity, further enhancing tumor cell cytotoxicity. In *in vivo* experiments, this drug delivery system markedly induced tumor apoptosis, disrupted lipid metabolism and immune responses, and effectively overcame resistance in ICC.

The complexity of the TME suggests that even minor regulatory alterations can profoundly affect immune cell function. Within this complex TME, metabolic reprogramming of tumor cells impairs antigen presentation and recognition by immune cells, whereas intrinsic metabolic alterations in immune cells modulate their functional activity. This bidirectional metabolic crosstalk ultimately remodels the tumor immune microenvironment. Although SDT combined with metabolic reprogramming presents an innovative approach to reshaping the tumor immune microenvironment, it has several challenges to overcome. First, tumors may adapt to the metabolic stress induced by SDT via compensatory metabolic pathways, thereby promoting the recruitment of immunosuppressive cells and reestablishing an immunosuppressive TME. Second, excessively high levels of ROS generated by SDT may compromise the function and viability of tumor-infiltrating T cells, potentially inducing pyroptosis or apoptosis and exacerbating immune exhaustion. Furthermore, the absence of real-time monitoring tools for metabolic reprogramming and dynamic immune microenvironment changes during SDT limits the capacity for precise therapeutic regulation.

### 7.4 Multimodal therapy combined with correction of immunosuppressive TME

Multimodal therapy combines two or more treatment modalities (surgery, chemotherapy, radiotherapy, gene therapy, SDT, PDT, PTT and immunotherapy), effectively minimizing side effects while significantly enhancing therapeutic efficacy 116. Previous studies 117 have shown that an integrated multimodal “photo-sonodynamic therapy and immunotherapy” platform—combining SDT, PDT, and immune activation—can treat tumors *in situ* while concurrently preventing recurrence after surgical resection. Xu *et al.* 118 developed a microalgae-MOF hybrid system by conjugating MOF nanoparticles with Chlorella vulgaris. Chl-MOF generates O₂ via photosynthesis, alleviating tumor hypoxia. Under laser and ultrasound irradiation, Chl-MOF generates ROS, enhancing photo-sonodynamic effects and inducing tumor cell apoptosis. Owing to the high mobility and bioactive content of Chl, compared with MOF alone, Chl-MOF accumulates 3.3-fold more efficiently at deep tumor sites and promotes immune cell activity and infiltration 119. Chl-MOF reverses the immunosuppressive TME by enhancing NK cell cytotoxicity and dendritic cell antigen presentation, thereby eliciting robust antitumor immunity. Further studies demonstrated that Chl-MOF significantly suppressed breast tumor growth *in vitro* and *in vivo* via synergistic SDT, PDT, and immunotherapy, highlighting its potential as a cancer theranostic platform.

The μ-oxydipyrrole complex ([Fe(III)PpIX]_2_O) located on the cell walls (CWs) of *Porphyromonas gingivalis* (Pg) exhibits remarkable peroxidase (POD)-mimetic and SDT activities 120. Yang *et al.* integrated multiple therapeutic modalities to remodel the immunosuppressive TME 121. They engineered *Pg* cell walls into nanoscale CW vesicles (CWVs) enriched with LPS as biomimetic sonosensitizers for SDT. The CWVs were further loaded with doxorubicin to construct DOX@CWV (Figure 7D). Under ultrasound irradiation, DOX@CWV generates cytotoxic ROS, inducing ICD. Meanwhile, its intrinsic POD-like activity catalyzes H₂O₂ decomposition to generate O₂ *in situ*. This process consumes intracellular GSH, reducing ROS scavenging and enhancing the efficacy of SDT. Ultrasound further accelerates DOX, enabling controlled drug delivery at tumor sites. The released DOX induces tumor cell apoptosis, synergistically enhancing the efficacy of SDT and reducing systemic toxicity. In addition, dying tumor cells release DAMPs that trigger robust antitumor immune responses. These responses include DC maturation, effector T-cell differentiation, and M1 macrophage polarization, which collectively suppress tumor growth and metastasis. Taken together, these findings suggest that DOX@CWV suppresses tumor growth via combined SDT and chemotherapy, eliciting synergistic antitumor immunity through the integration of bacterial immunotherapy and SDT.

Although SDT has shown substantial immune-activating potential when combined with other therapeutic modalities and can effectively alleviate the immunosuppressive TME, its clinical translation still faces multiple challenges. Current limitations include not only the *in vivo* accumulation and metabolism of sonosensitizers, insufficient drug-loading efficiency of nanocarriers, uncontrolled ROS generation that may damage surrounding tissues, and adverse systemic effects caused by excessive immune activation, but also deeper challenges such as tumor heterogeneity, uneven nanoparticle distribution resulting from high interstitial fluid pressure in solid tumors that hinders effective penetration, and the dynamic plasticity of the TME. Excessive therapeutic intervention may further induce compensatory recruitment of immunosuppressive cells, leading to the reestablishment of an immunosuppressive TME. Therefore, future research should focus on achieving precise modulation of the TME while simultaneously developing advanced nanodelivery systems, in order to prevent treatment-induced compensatory feedback mechanisms.

## 8. SDT Sensitization Strategy - Enhance Cytotoxic Effects

### 8.1 SDT combination chemotherapy enhances cytotoxic effects

Long-term chemotherapy may lead to growth retardation, immune dysfunction, and even the development of secondary malignancies 5. Gong *et al.* 122 proposed a dual-action strategy that combines low-dose chemotherapy with enhanced SDT to reduce chemotherapy-related toxicity and address interpatient variability. Barium titanate nanoparticles were used as sonosensitizers, with ultrasound enhancing chemotherapeutic drug uptake. Concurrently, 5-FU promoted nanoparticle internalization, and the combined treatment markedly inhibited tumor cell proliferation. Patient-derived tumor organoids revealed interpatient variability, highlighting the potential for personalized treatment. Notably, comparable antitumor efficacy to that of high-dose chemotherapy was achieved in drug-resistant organoids.

**Figure 8:**
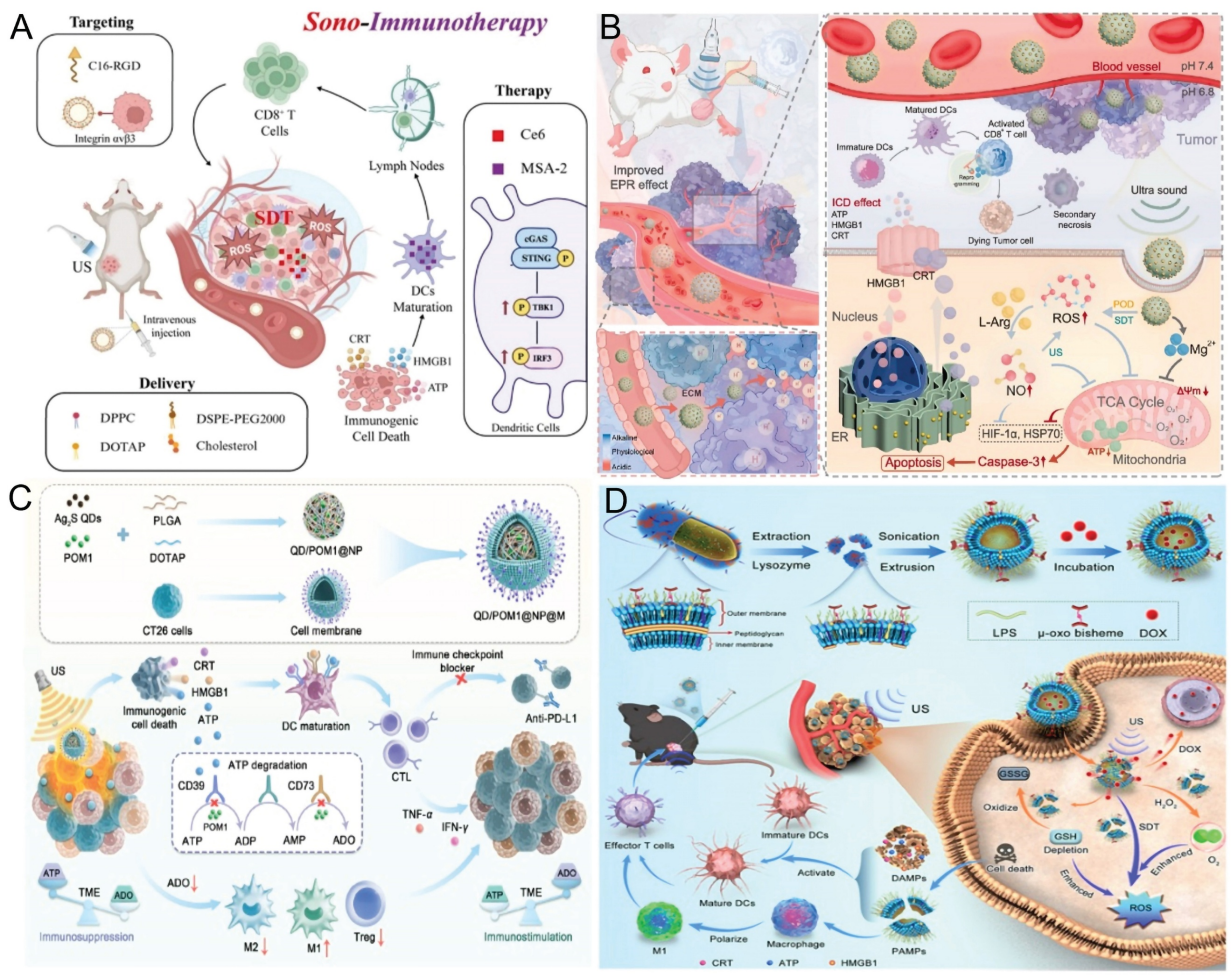
Enhance cytotoxic effects A) Schematic illustration of IR780@LDs-Fe3O4/OA-mediated cascade-targeted SDT reversing Dox resistance in breast cancer. Adapted with permission from 124, *copyright 2024 American Chemical Society* B) Schematic illustration of the preparation and treatment mechanisms of EXO@CAT NVs. Adapted with permission from 133, *copyright The Author(s).* C) A scheme to show the preparation of CMC heterojunctions for tumor-specific cuproptosis-enhanced SDT/CDT/immunotherapy. Adapted with permission from 146*, copyright 2025. The Author(s).* D) Schematic illustration of PMC-mediated SDT enhancement in synergy with RT for tumor cell elimination. Adapted with permission from 152, *copyright 2024 Jiang et al*.

Recent studies have focused on exploiting SDT-generated ROS to disrupt intracellular organelles, such as damaging mitochondria or compromising lysosomal membranes 123, 124. Lysosomes can sequester lipophilic, weakly basic chemotherapeutic drugs such as DOX, which can passively diffuse across cell membranes at physiological pH. In acidic lysosomes, protonation of the amino group prevents drug diffusion, resulting in lysosomal trapping 125-127. This trapping reduces effective drug accumulation and compromises chemotherapy efficacy. To overcome multidrug resistance (MDR) in breast cancer, Shi *et al.* 124 used endogenous lipid droplets as carriers targeting lysosomes and mitochondria. Adipocyte-derived LDs encapsulated IR780 and OA-coated magnetic Fe_3_O_4_ nanoparticles to form IR780@LDs-Fe_3_O_4_/OA composites (Figure 8A). Magnetic Fe_3_O_4_ nanoparticles enable tumor accumulation under a 0.3 T magnetic field. Owing to their membrane-mimicking lipid composition, LDs are readily internalized by tumor cells. Confocal imaging confirmed their predominant localization in lysosomes and mitochondria. Upon ultrasound excitation, IR780 generates ROS that disrupt lysosomal membranes and release trapped DOX. This process disrupts the mitochondrial membrane potential, inhibits ATP production, and suppresses P-gp efflux, promoting intracellular DOX accumulation. Under ultrasonic cavitation and acidic lysosomal conditions, Fe_3_O_4_ partially dissolves to release Fe^2+^, which catalyzes Fenton reactions with H_2_O_2_, generating highly toxic •OH. This cascade enables effective and safe combination therapy that integrates SDT with chemotherapy.

Although SDT-enhanced chemotherapy has shown significant advantages in both *in vitro* and *in vivo* tumor treatment studies, the encapsulation efficiency and *in vivo* stability of nanocarriers for chemotherapeutic drugs still require optimization to prevent premature drug release. Furthermore, critical concerns—including the dual cytotoxicity of chemotherapy drugs and ROS potentially harming normal tissues, as well as the long-term retention and associated toxicity of carrier materials *in vivo*—require systematic evaluation and further mitigation.

### 8.2 SDT- synergistic ferroptosis

Ferroptosis is a recently identified iron-dependent form of programmed cell death that is typically initiated by excessive intracellular ROS production. This results in significant lipid peroxidation (LPO) of cellular membranes, a process often facilitated by GSH depletion, which subsequently inhibits glutathione peroxidase 4 (GPX4) activity 128, 129. Recent studies 130-132 have further enhanced the synergistic efficacy of SDT and ferroptosis in tumor therapy by improving the targeting capabilities of nanoparticles. Wu *et al.* 133 loaded tumor-derived exosomes (EXO@CAT NVs) with the sonosensitizer TCPP via electroporation (Figure 8B). Owing to their catalase activity, EXO@CAT NVs alleviate tumor hypoxia after cellular internalization. Under ultrasound irradiation, TCPP generates large amounts of ¹O₂, enhancing the efficacy of SDT. Concurrently, ACSL4 promotes lipid peroxidation, increasing ferroptosis susceptibility. *In vivo* studies confirmed that oxygen-enriched SDT combined with ACSL4 significantly increased ferroptosis sensitivity, resulting in tumor growth inhibition.

Zhao *et al.* 134 developed a cysteine-responsive nanoscale platform (Aza-BD@PC NPs) assembled from Aza-BD-modified DSPE-PEG and phenyl chlorothiocarbonate. After internalization by GBM cells, Aza-BD@PC NPs undergo S-N bond cleavage, depleting cysteine, generating H₂S, and disrupting redox balance and glycolysis. These effects collectively promote ferroptosis, induce DNA damage, and inhibit GBM cell proliferation. Moreover, the released Aza-BD generates large amounts of ¹O₂ under ultrasound, enabling effective SDT. *In vivo* studies in GL261 tumor models confirmed that combining gas therapy with ferroptosis synergistically enhanced the efficacy of SDT.

### 8.3 SDT-synergistic cuproptosis

The primary mechanism of cuproptosis involves the intracellular accumulation of copper ions, which directly bind to the acylated components of the tricarboxylic acid (TCA) cycle. This interaction induces the aggregation of acylated proteins and the loss of iron‒sulfur cluster proteins, thereby disrupting TCA cycle-associated metabolic pathways. The resulting metabolic imbalance and proteotoxic stress ultimately lead to cell death 135. Unlike conventional apoptosis, cuproptosis can induce ICD through the release of DAMPs, including HMGB1, ATP, and CRT, thereby promoting DCs maturation and tumor antigen presentation 136. The activated DCs subsequently present tumor antigens to CD8⁺ T cells and deliver costimulatory signals and cytokines, thereby driving CD8⁺ T-cell proliferation and differentiation into effector CTLs 137. These immune-activating processes synergize with SDT to amplify ICD, further enhancing CD8⁺ T cell proliferation and cytotoxic function, increasing intratumoral T cell infiltration 138, and concurrently reducing Tregs and myeloid-derived suppressor cells (MDSCs), thereby effectively reversing the immunosuppressive tumor immune microenvironment 139. Multiple studies 140-142 have demonstrated that combining SDT with cuproptosis effectively eradicates primary tumors and suppresses tumor metastasis. Zhong et al. 143 developed two-dimensional copper-based MOF sonosensitizers (CM) via coordination between copper ions and dimethylimidazole. CM can effectively harness the high pressure generated by ultrasonic cavitation to activate its piezoelectric properties, thereby enhancing ROS production through band bending and band gap reduction and promoting electron‒hole separation. Additionally, Cu^2+^ in CM reacts with GSH to produce Cu^+^ and oxidized glutathione (GSSG) 144, 145, effectively depleting GSH and promoting Fenton-like reactions. These reactions catalyze the conversion of H_2_O_2_ into •OH, further amplifying oxidative damage. Copper ions in CM also enter the TCA cycle, inducing abnormal oligomerization of dihydrolipoamide S-acetyltransferase (DLAT) and the loss of iron‒sulfur cluster proteins, ultimately triggering cuproptosis. Cao *et al.* 146 constructed a Z-type heterojunction (CMC) by *in situ* coating a Cu-MOF protective layer onto a Cu_2_O surface. The CMC remains stable under normal physiological conditions but selectively degrades within the TME upon US irradiation, releasing Cu⁺ and Cu²⁺ ions to achieve cascade-amplified ROS production. Additionally, the released Cu^+^ induces tumor-specific cuproptosis. Additionally, the CMC heterojunction markedly enhances ICD by increasing ROS levels and inducing cuproptosis through Cu^+^-mediated DLAT oligomerization and mitochondrial dysfunction. This synergistic mechanism enables complete elimination of primary tumors and near-total suppression of distant metastases (Figure 8C). Compared with other copper-based nanomaterials, Cu_2_O demonstrates superior SDT and Fenton-like reactivity, owing to its narrow bandgap and the presence of Cu 147. Geng *et al.* 141, 148 systematically validated and developed a TME-responsive nanoplatform (GQD/Cu₂₋ₓSe) that undergoes GSH-triggered degradation, resulting in the controlled release of graphene quantum dots (GQDs), copper ions, and selenium ions. The released copper ions amplify ROS generation via cascade reactions, thereby inducing tumor-specific cuproptosis and triggering ICD. Concurrently, selenium ions directly promote DCs maturation, thereby potentiating antitumor immunotherapy. Meanwhile, GQDs inhibit topoisomerase I/II activity by binding to the major groove of DNA, leading to DNA damage-mediated apoptosis and enhanced chemotherapeutic efficacy. Collectively, these mechanisms enable a synergistic SDT-based therapeutic strategy that integrates cuproptosis, chemotherapy, and immunotherapy.

Although metal-mediated cell death combined with CDT and other therapeutic modalities has shown great promise in enhancing SDT-induced ROS generation and achieving “1+1>2” antitumor effects, several key challenges remain to be addressed. First, current sonosensitizer development primarily emphasizes improving ROS generation efficiency while overlooking biocompatibility and the potential *in vivo* accumulation of metal ions, which may result in long-term toxicity. Second, the absence of an efficient and precise delivery system results in the nonspecific distribution of sonosensitizers within normal tissues, potentially causing irreversible damage to healthy cells during SDT. Third, visually monitoring the intratumoral accumulation of sonosensitizers during SDT procedures remains challenging. Future research should not only aim to increase ROS production but also improve the targeting specificity, biocompatibility, and metabolic safety of sonosensitizers, while enabling real-time visualization throughout the entire treatment process.

### 8.4 SDT combined radiotherapy enhances cytotoxic effects

Radiation therapy (RT) is a cancer treatment modality that employs X-rays to inhibit cell proliferation, generate highly cytotoxic ·OH radicals, and induce double-stranded DNA damage 149, 150. Research 151 has demonstrated that combining SDT with RT can synergistically increase antitumor efficacy. Yi *et al.* 152 synthesized MnCO₃ NPs via a reverse microemulsion method and coated them with platelet membranes to construct a biomimetic nanoplatform (PMC) (Figure 8D). This platform efficiently targeted deep tumor regions enriched in cancer stem cells (CSCs), enabling potent synergistic SDT-RT therapy. Following intravenous administration, the accumulation of PMC is increased in CSCs. Under ultrasound activation, PMC produces ^1^O_2_ and •OH, thereby enabling the selective elimination of CSCs and other tumor cells. When combined with low-dose RT, this platform has a synergistic inhibitory effect on tumor growth. *In vitro* and *in vivo* experiments demonstrated that the combination therapy of PMC, ultrasound, and 4 Gy RT had pronounced synergistic antitumor effects without compromising biological safety.

To alleviate the hypoxic TME and enhance the therapeutic efficacy of SDT combined with RT, Liu *et al.* 153 developed oxygen-rich ICG@O_2_ nanobubbles with a core-shell architecture. These nanobubbles possess an oxygen-filled gaseous core and a lipid shell encapsulating the sonosensitizer ICG. Ultrasound triggers the disruption of nanobubbles, accelerating oxygen release and thereby improving tumor-specific oxygen delivery. Subsequently, SDT was carried out using therapeutic ultrasound irradiation, which activated the sonosensitizer to generate ROS, inducing apoptosis or necrosis and thereby markedly enhancing SDT efficacy. Following X-ray irradiation, the generated •OH causes additional DNA damage in tumor cells, effectively suppressing their proliferation. ICG@O_2_ nanobubbles facilitate targeted delivery of the sonosensitizer ICG and enable controlled oxygen release at tumor sites, alleviating hypoxia and enhancing ROS-mediated cytotoxicity.

Currently, the combination of SDT and RT has progressed to clinical trials, demonstrating both therapeutic potential and a favorable safety profile. A phase I clinical trial 154 investigated the combination of SDT and RT in 11 patients with brainstem gliomas (BSGs), who received SDT and RT following the administration of heme porphyrin. Among the 11 patients, 8 (72.7%) maintained stable disease, and 2 (18.2%) achieved partial remission. The median progression-free survival (PFS) of the patients was 9.2 months, while the median overall survival (OS) was 11.7 months. The combination of SDT and RT has demonstrated favorable safety and feasibility, along with promising preliminary therapeutic potential.

Although the use of SDT combined with RT has progressed through phase I clinical trials, several limitations remain. First, hematoporphyrin can be activated not only by ultrasound and radiation but also by light, resulting in photosensitivity. Therefore, strict light avoidance is necessary after treatment until the hematoporphyrin is metabolized to nonphototoxic levels, but the slow metabolism of hematoporphyrin in the blood necessitates an extended period of light avoidance after treatment, thereby limiting its clinical utility. Second, the optimal sequence for initiating SDT and RT after hematoporphyrin administration remains a subject of debate, as does the standardization of the treatment parameters for SDT and RT. Therefore, the development of sonosensitizers with high selectivity and favorable metabolic profiles, together with the establishment of standardized treatment parameters, remains a critical research priority for clinical translation in this field.

Although direct quantitative comparisons of therapeutic efficacy across different SDT-based combination strategies remain challenging because of variations in tumor models and treatment parameters, a qualitative comparison in terms of mechanism, complexity, and potential safety concerns can provide valuable insights into their respective advantages and limitations (Table 2).

## 9. SDT Sensitization Strategy - Precision Controlled-Release ROS

**Figure 9:**
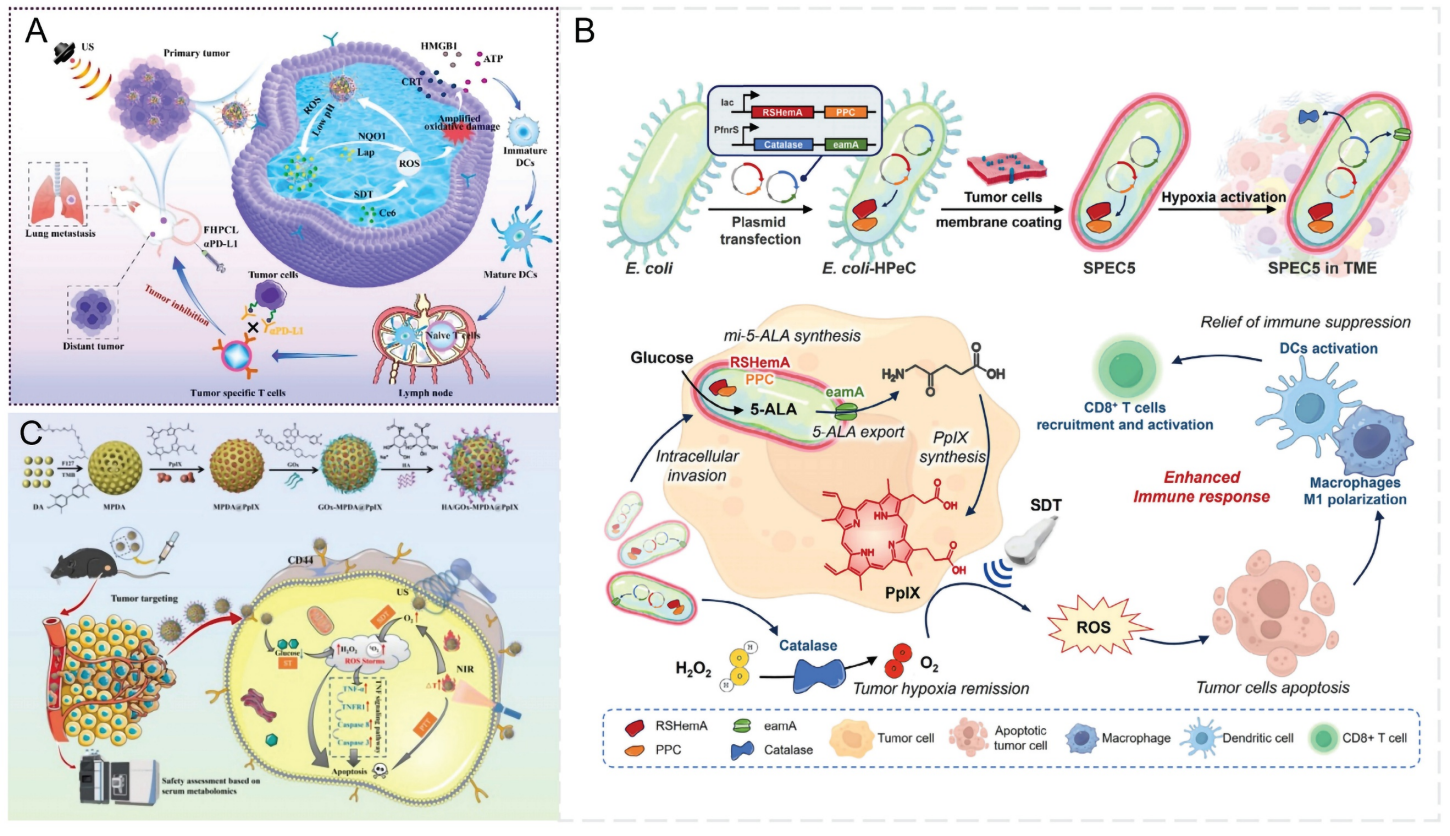
Precision controlled-release ROS A) Preparation process of FHPCL NPs and schematic diagram of FHPCL NPs-enhanced SDT combined with αPD-L1 synergistic immunotherapy. Adapted with permission from 156, *copyright 2025 Elsevier Ltd.* B) Preparation of the bacterial 5-ALA delivery system SPEC5 and its induction of enhanced antitumor SDT activation in immunotherapy processes. Adapted with permission from 161, *copyright 2025 Wiley-VCH GmbH.* C) Schematic diagram of synthesizing HGMP NPs and utilizing their active targeting of tumor cells in combination with SDT, PTT, and ST for tumor treatment. Adapted with permission from 172, *copyright 2025. The Author(s)*.

To enhance the tumor targeting ability of nanoparticles, precise, controlled drug release within tumor regions and efficient ROS generation is necessary. Multiple studies 34, 155 have demonstrated that nanoparticles surface- modified with tumor-specific ligands or hybridized with cell membrane mimics can efficiently target tumor cells and be internalized, thereby achieving selective accumulation within tumor regions. Zhu *et al.* 156 utilized Lap as an endogenous ROS amplifier and designed a pH/ROS dual-responsive nanoplatform (FHPCL NPs) coloaded with Lap and the sonosensitizer Ce6. After internalization by tumor cells, TK linkages, and the pH-sensitive hydrazone (HYD) linkages undergo cleavage under elevated ROS levels and acidic conditions, enabling the controlled release of Ce6 and Lap. Ultrasonic irradiation activates Ce6, further amplifying ROS generation, which promotes the decomposition of FHPCL NPs and mPEG-TK-Ce6, thereby initiating a self-amplifying oxidative cascade. This cascade amplification induces ROS bursts, exacerbates oxidative damage in tumor cells, and triggers pyroptosis, thereby generating potent ICD effects that promote DCs maturation and CTLs infiltration. Furthermore, the combination of FHPCL nanoparticles with ultrasound irradiation enhanced the therapeutic efficacy of the αPD-L1 antibody, effectively inhibiting both primary and metastatic tumor growth in 4T1-bearing mice while inducing long-term immune memory. This strategy enables precise, controlled delivery of SDT combined with immunotherapy within tumor cells (Figure 9A).

*In situ* biosynthesis strategies utilizing bacterial functional molecules 157 eliminate the need for systemic administration 158, minimize off-target effects 159 and achieve high local concentrations of therapeutic payloads within the TME 160. This approach promotes intratumoral accumulation of sonosensitizers and enables their selective production directly within tumor cells. Yang *et al.* 161 developed a probiotic-based precision-controlled 5-ALA delivery system (SPEC5) (Figure 9B), which exploits the natural tumor-targeting ability of engineered *Escherichia coli* 162-164 in combination with synthetic biology techniques. This system enables the bacteria to continuously synthesize 5-ALA within the TME and selectively induce PpIX production under hypoxic conditions. As a result, it effectively overcomes the limitations of conventional 5-ALA delivery, including poor tumor selectivity and systemic off-target effects. Although bacteria can be rapidly cleared following SDT, systemic administration of engineered bacteria still carries potential biosafety risks, necessitating further attenuation modifications to improve their safety profile. Furthermore, although SDT-induced immune activation holds considerable promise for immunotherapy, encapsulating tumor cell membranes may decrease nanomaterial clearance while inadvertently facilitating tumor immune evasion. Another critical challenge is the extracellular diffusion of sonosensitizers, which can diminish the tumor selectivity of SDT and cause off-target effects. Consequently, novel strategies are needed to enhance intracellular retention of sonosensitizers or to more effectively control their extracellular distribution.

Owing to the abnormal metabolic behavior of cancer cells, which consume glucose more actively than normal cells to sustain rapid proliferation 165-167, starvation therapy (ST) mediated by glucose oxidase (GOx) catalyzes the oxidation of glucose into gluconic acid and H_2_O_2_, thereby effectively restricting the energy supply to tumor cells and inhibiting their proliferation 168, 169. Previous studies 170, 171 have shown that synergistic strategies combining SDT with multiple therapeutic modalities, such as ST, possess considerable antitumor potential. Wen *et al.* 172 designed a core-shell nanoparticle, HA/GOx-MPDA@PpIX (HGMP NPs), by surface functionalizing the photothermal agent mesoporous polydopamine (MPDA) to serve as a core carrier for PpIX, with further surface modification by GOx and HA for synergistic antitumor therapy. HGMP NPs actively target tumor sites and accumulate specifically via HA-mediated interaction with CD44 receptors. Under 808 nm NIR and US irradiation, HGMP NPs display PTT and selective SDT activity. NIR-triggered PTT efficiently eliminates tumor cells, increases oxygen availability at the tumor site, enhances SDT efficacy, and further amplifies ROS generation (Figure 9C). SDT overcomes the limited tissue penetration of PTT, facilitating more comprehensive tumor eradication.

Although multimodal therapies combining SDT with other modalities have shown considerable therapeutic potential, they continue to face several critical challenges. First, achieving precise spatiotemporal alignment of different therapeutic components at the tumor site is challenging, limiting the full realization of synergistic effects. Second, the mechanisms governing interactions among multiple treatment modalities remain incompletely understood. Disrupted cascade reactions, interference between ultrasound and thermal effects, and variations in treatment sequence may lead to unstable synergistic outcomes. Furthermore, the long-term biosafety of nanomaterials requires thorough evaluation, and the potential risk of excessive immune activation must be carefully considered.

## 10. Summary and Outlook

Although SDT combined with other therapeutic regimens has demonstrated significant synergistic efficacy and broad potential in cancer treatment, several challenges remain that limit its clinical translation: First, the paucity of pharmacokinetic and biocompatibility data for nanoscale sonodynamic agents hinders the rational optimization of targeted modification strategies; Second, current strategies aimed at enhancing ROS generation—often in combination with CDT, PDT, and RT—lack standardized ultrasonic parameter frameworks; Finally, quality control challenges during good manufacturing practice (GMP)-compliant production further increase the complexity of achieving reliable TME-specific delivery.

In recent years, to enhance the tumor-targeting ability of nanoparticles, researchers have developed various advanced strategies such as surface modification with tumor-specific ligands for targeted recognition 49, 52, 65, 98, 132, 152, 172, cell membrane camouflage to improve biocompatibility and tumor-homing ability 5, 56, 113, 161, and the use of magnetic nanoparticles to achieve precise accumulation in tumor regions under external magnetic guidance 124. In addition, to overcome the challenge of high interstitial pressure in solid tumors, which impedes drug delivery and reduces therapeutic efficacy, the use of SDT in combination with other therapies has attracted increasing attention. Owing to their structural properties, nanoparticles can accumulate in tumor regions and be internalized by tumor cells. Researchers have developed pH-responsive drug release systems that selectively release payloads in the acidic TME 56, 64, 146, 156. Moreover, payload release can be precisely controlled through the cleavage of chemical bonds triggered by ROS generated from ultrasound-activated sonosensitizers 98, 156, thereby enhancing therapeutic efficacy. However, the dense extracellular matrix and abnormal vascular structure within tumors continue to impede the effective intratumoral distribution of nano-sonosensitizers, particularly in the tumor core, where achieving adequate accumulation is essential for optimal therapeutic outcomes. In addition, tumors display pronounced regional heterogeneity in blood flow, oxygenation, and receptor expression 173, which further hinders the uniform targeting and delivery of sensitizers to all tumor cells 174, 175. Therefore, to achieve precise and comprehensive control of nanoparticle release within tumor regions, future research should focus on optimizing targeted nanoparticle modifications and developing loading platforms engineered according to specific TME characteristics. At present, comprehensive pharmacokinetic (PK) data are lacking, and consequently, there is no rational basis for the targeted design and modification of nanoparticles. Moreover, the effects of such modifications on key nanomaterial PK parameters—including half-life, volume of distribution, and clearance—have not been systematically elucidated. Therefore, to achieve precise and spatially controlled drug release within tumor regions, future studies should not only emphasize rational nanoparticle targeting strategies and the development of TME-responsive loading platforms, but also systematically evaluate the biosafety of sonosensitizers.

SDT-induced antitumor immunity is significantly constrained by insufficient ROS production 176, largely due to the poor stability and low bioavailability of conventional organic sonosensitizers, as well as the limited ROS generation efficiency of classical inorganic sonosensitizers 9. Moreover, most current sonosensitizers are derived from photosensitizers and thus exhibit inherent drawbacks, including nonspecific biodistribution, inadequate tumor accumulation, and undesirable phototoxicity 13. Recognizing these limitations, researchers have increasingly focused on developing advanced sonosensitizers capable of enhancing ROS generation. Promising candidates include piezoelectric materials, defective semiconductors, hybrid-assembled polymers with narrow band gaps, and novel heterojunction-engineered sonocatalysts 177. Ultrasound stimulation induces electron‒hole pair separation within piezoelectric materials, generating an internal electric field that drives electrons and holes to migrate toward opposite surfaces, where they participate in redox reactions to produce abundant ROS 36, as demonstrated in materials such as BaTiO_3_ 98, 122, 178. Defective semiconductor materials under ultrasonic irradiation not only suppress electron‒hole recombination through oxygen vacancies that act as electron traps but also ncrease the charge separation efficiency. Compared with conventional organic sonosensitizers, these defect sites further increase the adsorption of water molecules and oxygen, thereby promoting heterogeneous catalytic reactions and enabling substantially higher ROS generation rates and yields 179, 180. Hybrid-assembled polymers with narrow band gaps 59, 118, 143, 181, as well as novel heterojunction-based sonocatalysts 65, 146, have also been shown to possess strong ROS-generating potential. However, the *in vivo* accumulation behavior of nanomaterials and the potential risk that excessive ROS may inflict damage on normal tissues still warrant careful consideration. Therefore, in the development of next-generation sonosensitizers aimed at increasing ROS generation, careful attention must be given to their biosafety, ensuring an optimal balance between therapeutic potency and biocompatibility. Furthermore, the translation of nanopharmaceuticals from laboratory-scale synthesis to GMP-compliant production faces dual challenges related to batch-to-batch consistency and TME-responsive stability. First, minor variations in nanoscale synthesis processes can result in significant variability in critical physicochemical properties, including particle size distribution and drug loading capacity, thereby impairing tumor targeting efficiency and tissue penetration; second, stimulus-responsive behaviors dependent on TME-specific cues are often difficult to reproducibly maintain under GMP manufacturing conditions.

SDT-induced antitumor immunity is also constrained by the TME 17, where hypoxia and high antioxidant capacity suppress ROS generation. However, combining SDT with other therapeutic modalities can partially modulate these TME characteristics, thereby enhancing the efficacy of SDT. To address hypoxia, strategies include the delivery of oxygen-loaded nanoparticles to the tumor site 153. The decomposition of H_2_O_2_ by POD during CDT not only alleviates hypoxia and supplies O_2_ for SDT-mediated ^1^O_2_ generation, but also promotes further H_2_O_2_ production via CDT. This cascade enhances the formation of cytotoxic •OH, increases overall ROS levels, and achieves synergistic tumor cell killing. When combined with SDT and CDT, the nanoplatform can also deliver hypoxia-inducible factor-targeting siRNA and chemotherapeutic drugs, further alleviating hypoxia and enhancing tumor cell cytotoxicity. Another approach is the use of engineered bacteria, which can exploit the hypoxic TME to selectively localize nanoparticles, thereby achieving targeted accumulation within tumor tissues. To address the high antioxidant TME, strategies include incorporating metal ions with GSH-oxidase activity into nanoparticles. CDT not only generates cytotoxic •OH but also consumes intracellular GSH, further weakening the antioxidant defense. Additionally, nanoplatforms can deliver drugs such as siRNAs that inhibit GSH synthesis, thereby further depleting GSH, reducing ROS scavenging, and enhancing SDT efficacy. This GSH depletion also promotes ferroptosis in tumor cells, contributing to improved therapeutic outcomes. The low pH of the TME generates an acidic gradient that can be exploited to generate an endogenous electric field that guides nanoparticles to tumor sites, and acid-responsive nanoparticles can be designed to release their payloads via cleavage of pH-sensitive chemical bonds in highly acidic environments. Payloads may include chemotherapeutic drugs and siRNAs targeting hypoxia, antioxidant defenses, or ROS resistance, as well as inhibitors of energy and lipid metabolism, enabling synergistic SDT with enhanced antitumor efficacy. To relieve immunosuppression, SDT can be combined with other therapies using nanoplatforms that carry immune checkpoint inhibitors or other immunomodulatory drugs. These nanoplatforms accumulate in the TME and release their payloads either passively within the TME or upon ultrasound stimulation. Upon irradiation, the sonosensitizer generates large amounts of ROS, directly inducing ICD, promoting immune cell maturation, enhancing antigen presentation, increasing T cell infiltration, and ultimately remodeling the tumor immune microenvironment to strengthen antitumor immunity. This also offers a novel strategy for developing future therapeutic strategies. Multimodal treatments combined with modulation of the TME can enhance antitumor efficacy and improve overall cancer therapy outcomes. Moreover, selectively leveraging certain TME characteristics may further facilitate therapeutic effects, supporting more effective tumor eradication. However, a critical barrier to clinical translation of SDT is the absence of standardized ultrasonic parameter frameworks. Specifically, ultrasonic frequency and intensity directly modulate ROS generation by regulating acoustic cavitation phenomena, wherein high-intensity ultrasound is typically applied for tumor ablation, whereas SDT necessitates moderate parameter settings to minimize injury to surrounding normal tissues. Particularly in combination therapy paradigms, precise optimization of ultrasonic parameters is essential, highlighting the urgent need to establish optimized parameter standards that balance therapeutic efficacy and biosafety.

While SDT can directly induce tumor cell death to some extent, combining SDT with other therapeutic strategies has become a current research focus. However, key challenges remain, including the synthesis of nanomaterials, efficient ROS generation, precise targeting of the complex TME, and controlled payload release within the TME to achieve the desired therapeutic effects. To enhance therapeutic efficacy, recent research has achieved substantial progress in three principal areas: (1) improving targeting efficiency through ligand modification strategies, including recognition of tumor cell surface receptors, biofilm encapsulation, and external force-guided delivery of magnetic or ultrasound-responsive nanomaterials; (2) intelligent stimulus-responsive design that exploits tumor microenvironment-specific features, such as low pH, elevated GSH levels, and ROS, to trigger nanomaterial activation, enabling precise release of therapeutic agents or sonosensitizers and cascaded ROS amplification; and (3) multimodal synergistic strategies that integrate CDT, PDT, and immunotherapy to achieve complementary effects, enhance ROS generation, and induce tumor cell death through multiple mechanisms. In the future, the integration of artificial intelligence-assisted sonosensitizer design, sonogenetics, and other emerging technologies, together with the development of real-time image-guided SDT feedback systems, is expected to enable more precise and efficient tumor treatment. Nevertheless, further fundamental investigations and well-designed clinical studies are needed to facilitate clinical translation.

## Figures and Tables

**Table 1:** Comparison table of organic and inorganic sonosensitizers

Material type	ROS yield and types	Biocompatibility	Stability	Piezoelectric property	Defect Engineering	Progress in transformation
Organic sonosensitizer	Medium, ¹O₂ more common	High	Low	Optional	Few	Preclinical stage
Inorganic sonosensitizer	High, ·OH more common	Medium	High	Common	Common	Laboratory stage

**Table 2:** Comparison of SDT combined with other therapies

Combined treatment	Main mechanism of action	Sensitization Strategy	Treatment of complexity	Potential side effect risks
SDT + Immunotherapy	SDT induces ICD, enhances antigen release and activates T cells. The response synergizes with immune checkpoint inhibitors.	Immune-suppression TME correction	High	Immune-related adverse reactions, inflammatory responses
SDT + Gas therapy	SDT triggers or enhances the release of gases, improving tumor blood flow, alleviating hypoxia and regulating the TME	Immune-suppression TME correction	Medium	Gas dose-related toxicity, systemic side effects
SDT + Metabolic reprogramming	SDT interferes with the metabolic pathways of tumor cells or immune cells, reducing immunosuppressive metabolic products.	Immune-suppression TME correction	High	Metabolic disorders and potential non-specific effects
SDT + Multimodal treatment	SDT works in conjunction with various treatment modalities, and regulates the TME and cellular stress responses through multiple pathways.	TME regulation + Cytotoxic effects	Higher	Cumulative toxicity, complex treatment regimens
SDT + Chemotherapy	SDT enhances the delivery and uptake of chemotherapy drugs, and amplifies ROS-induced DNA damage and cytotoxicity.	Cytotoxic effects	Medium	Chemotherapy-related systemic toxicity
SDT + Ferroptosis	SDT promotes lipid peroxidation and the accumulation of iron-dependent ROS, and induces the ferroptosis pathway	Cytotoxic effects	Medium	Toxicity related to oxidative stress
SDT + Cuproptosis	SDT interferes with mitochondrial metabolism and enhances copper ion-dependent protein toxicity stress	Cytotoxic effects	Medium - High	Toxicity related to metal ions
SDT + Radiotherapy	SDT enhances the generation of ROS and DNA damage induced by radiation, thereby increasing radiotherapy sensitivity.	Cytotoxic effects	High	Radiation injury, tissue inflammation

**Table 3:** Summary of SDT sensitization strategy

Sensitization Strategy	Core Strengths	Limitations	Applicable scenarios
Improvements to Sonosensitizers	Enhance ROS generation efficiency, enable real-time guidance of the SDT process, and intelligently respond to TME.	Achieving both biocompatibility and piezoelectric/acoustic performance remains challenging, and further validation is required for material stability and *in vivo* safety.	Fundamental SDT Research on Solid Tumors and Exploration of Integrated Imaging-Therapy Approaches
Relieve hypoxia TME	By alleviating tumor hypoxia through multiple pathways—including *in situ* oxygen production, catalytic decomposition of H₂O₂, improved tumor blood flow perfusion, and introduction of gene therapy—the efficiency of ROS generation and sensitivity to SDT are significantly enhanced.	Oxygen production and gene regulation effects are significantly influenced by tumor heterogeneity, and challenges remain in achieving efficient delivery and ensuring the biosafety of gene therapy.	Suitable for solid tumor models with high hypoxia, particularly those exhibiting poor response to oxygen-dependent therapies such as SDT/RT/PDT.
Antioxidant depletion	By depleting antioxidant substances such as GSH within tumor cells or introducing siRNA to inhibit the expression of antioxidant-related genes, the ROS scavenging capacity is weakened, thereby amplifying the cytotoxic effects of ROS generated by SDT.	The risk of inducing oxidative stress in normal tissues remains a key challenge, with selectivity and dose control still being critical issues.	Suitable for tumor types with strong antioxidant capacity and tolerance to single ROS therapy
Enhance ROS generation	By combining SDT with either CDT or PDT, synergistic amplification of ROS production is achieved. SDT enhances the Fenton/Fenton-like reaction efficiency of CDT or compensates for the limited light penetration of PDT in deep tissues, thereby significantly increasing overall ROS yield and tumor killing capacity.	Different therapeutic modalities exhibit strong dependence on the TME. For instance, CDT is constrained by H₂O₂ concentration and pH levels, while PDT is influenced by light exposure conditions. Combined strategies still face challenges in parameter matching and safety assessment during *in vivo* application.	Suitable for solid tumors where single SDT ROS generation is insufficient, particularly deep-seated or highly heterogeneous tumor models.
Correct immunosuppressive TME	SDT can induce anti-tumor immune responses by activating the innate immune system, and when combined with immunotherapy, gas therapy, or metabolic reprogramming strategies, it alleviates immunosuppressive TME at multiple levels, enhancing immune cell infiltration and effector functions.	Different combination strategies exhibit significant variations in their mechanisms of action, with immune regulatory effects being substantially influenced by tumor type and individual variability. The stability of therapeutic efficacy still requires systematic validation.	For tumor types with significant immunosuppression or limited response to single-agent immunotherapy, as a functional extension strategy for PDT.
Enhances Cytotoxic Effects	Through the synergistic combination of combined chemotherapy, radiotherapy, and programmed cell death pathways such as ferroptosis and cuproptosis coupled with SDT, a multi-mechanism approach achieves deep tumor cell killing while reducing the risk of drug resistance.	The combined therapy regimen is complex, with significant challenges in dose and timing control, necessitating a systematic assessment of potential toxic side effects.	Suitable for solid tumor models characterized by high invasiveness, susceptibility to drug resistance, or elevated risk of recurrence.
Enhances tumor targeting ability	Enhance the enrichment efficiency of sonosensitizers at tumor sites through ligand modification, TME responsiveness, or physical targeting, thereby reducing damage to normal tissues.	The stability and *in vivo* distribution of targeted ligands remain influenced by multiple factors, and clinical translation remains uncertain.	Suitable for solid tumors with high precision treatment requirements, especially those adjacent to vital organs.

## Data Availability

We confirm that all methods were carried out in accordance with relevant guidelines and regulations, and that all necessary approvals were obtained for accessing and using public datasets.

## Abbreviations

5-ALA: 5-aminolevulinic acid
ACT: adoptive T-cell therapy
ADCC: antibody-dependent cell-mediated cytotoxicity
ADO: adenosine
ATP: adenosine triphosphate
BSGs: brainstem gliomas
CDs: carbon dots
CE: cholesterol esters
CRT: calreticulin
CSCs: cancer stem cells
CSDT: Chemo-sonodynamic synergistic therapy
CTLs: cytotoxic T lymphocytes
DAMPs: damage-associated molecular patterns
DCs: dendritic cells
DNA: deoxyribonucleic acid
ECM: extracellular matrix
GMP: Good Manufacturing Practice
GPX4: glutathione peroxidase 4
GQDs: graphene quantum dots
GSH-ox: glutathione oxidase
GSSG: oxidized glutathione
HIF-1α: hypoxia-inducible factor-1α
HMGB1: high-mobility group box 1
HMME: hematoporphyrin monomethyl ether
HSPs: heat shock proteins
ICB: immune checkpoint blockade
ICD: immunogenic cell death
ICG: indocyanine green
LIFU: low-intensity focused ultrasound
LPO: lipid peroxidation
MDR: multidrug resistance
MDSCs: myeloid-derived suppressor cells
MnO2: Manganese dioxide
MOF: metal-organic framework
NK: natural killer
NO: nitric oxide
OS: overall survival
PD-L1: programmed death-ligand 1
PDT: photodynamic therapy
PFOB: perfluorooctyl bromide
PFS: progression-free survival
PIT: photoimmunotherapy
PK: pharmacokinetic
PTT: photothermal therapy
RNA: ribonucleic acid
ROS: reactive oxygen species
RT: Radiation therapy
SDT: Sonodynamic therapy
SPDT: sono-piezoelectric dynamic therapy
ST: starvation therapy
TAAs: tumor-associated antigens
TCA: tricarboxylic acid
TME: tumor microenvironment
Tregs: regulatory T cells

## References

[1] Yumita N, Nishigaki R, Umemura K, Umemura S (1989). Hematoporphyrin as a sensitizer of cell-damaging effect of ultrasound. Jpn J Cancer Res.

[2] Chan YT, Zhang C, Wu J, Lu P, Xu L, Yuan H (2024). Biomarkers for diagnosis and therapeutic options in hepatocellular carcinoma. Mol Cancer.

[3] Rose TL, Kim WY (2024). Renal Cell Carcinoma: A Review. JAMA.

[4] Letai A, de The H (2025). Conventional chemotherapy: millions of cures, unresolved therapeutic index. Nat Rev Cancer.

[5] Chen S, Ma T, Wang J, Liang S, Liao H, Tan W (2024). Macrophage-derived biomimetic nanoparticles enhanced SDT combined with immunotherapy inhibited tumor growth and metastasis. Biomaterials.

[6] Peng L, Sferruzza G, Yang L, Zhou L, Chen S (2024). CAR-T and CAR-NK as cellular cancer immunotherapy for solid tumors. Cell Mol Immunol.

[7] Melero I, de Miguel Luken M, de Velasco G, Garralda E, Martin-Liberal J, Joerger M (2025). Neutralizing GDF-15 can overcome anti-PD-1 and anti-PD-L1 resistance in solid tumours. Nature.

[8] Beckers C, Pruschy M, Vetrugno I (2024). Tumor hypoxia and radiotherapy: A major driver of resistance even for novel radiotherapy modalities. Semin Cancer Biol.

[9] Yang Y, Wang N, Yan F, Shi Z, Feng S (2024). Metal-organic frameworks as candidates for tumor sonodynamic therapy: Designable structures for targeted multifunctional transformation. Acta Biomater.

[10] Wang XB, Liu QH, Wang P, Tang W, Hao Q (2008). Study of cell killing effect on S180 by ultrasound activating protoporphyrin IX. Ultrasonics.

[11] Fang L, Zeng W, Liu Y, Miao Y, Lu C, Xu Z (2025). Ultrasound-Responsive Lipid Nanosonosensitizers with Size Reduction and NO Release: Synergistic Sonodynamic-Chemo-Immunotherapy for Pancreatic Tumors. Angew Chem Int Ed Engl.

[12] Dou P, Chen L, Xie Y, Xu W, Zhang X, Deng X (2025). A blood-brain barrier-penetrating nanoreactor for tumor microenvironment modulation, precise MR imaging and synergistic therapy of glioma. Int J Pharm X.

[13] Gong Z, Dai Z (2021). Design and Challenges of Sonodynamic Therapy System for Cancer Theranostics: From Equipment to Sensitizers. Adv Sci (Weinh).

[14] Zhu Y, Wang D, Du C, Wu T, Wei P, Zheng H (2025). Ruthenium Single-Atom Nanozyme Driven Sonosensitizer with Oxygen Vacancies Enhances Electron-Hole Separation Efficacy and Remodels Tumor Microenvironment for Sonodynamic-Amplified Ferroptosis. Adv Sci (Weinh).

[15] Shan Q, Li R, Ying B, Zhu W, Wu X, Xu S (2025). Organic Sonosensitizers-based SDT with enhanced ROS generation. Ultrason Sonochem.

[16] Beishenaliev A, Loke YL, Lin CY, Goh SJ, Seema J, Huang Y (2025). Ultrasmall Gold Nanoparticles as "Three-in-One" Enzyme-Mimicking Nanocatalysts for Combined Sonodynamic/Catalytic Therapy in Breast Cancer. ACS Appl Mater Interfaces.

[17] Xu M, Wang P, Sun S, Gao L, Sun L, Zhang L (2020). Smart strategies to overcome tumor hypoxia toward the enhancement of cancer therapy. Nanoscale.

[18] Hsu PH, Almutairi A (2021). Recent progress of redox-responsive polymeric nanomaterials for controlled release. J Mater Chem B.

[19] Bai Q, Wang M, Wang K, Liu J, Qu F, Lin H (2024). CuPc-Fe@BSA nanocomposite: Intracellular acid-sensitive aggregation for enhanced sonodynamic and chemo-therapy. J Colloid Interface Sci.

[20] Zheng X, Pang L, Wang Y, Zhao Q, Pan G, He X (2025). Ultrasound-activated bimetallic PtRu alloy nanozymes for synergistic sonodynamic and chemodynamic therapy of multidrug-resistant bacterial infection. Mater Today Bio.

[21] He X, Wang Z, Mao B, Lin H, Jin X, Du M (2025). Trimetallic Pt-Pd-Au alloy nanozymes for multimodal synergistic therapy to overcome deep-seated drug-resistant infections via ROS cascade. Bioact Mater.

[22] Jiang Z, Xiao W, Fu Q (2023). Stimuli responsive nanosonosensitizers for sonodynamic therapy. J Control Release.

[23] Son S, Kim JH, Wang X, Zhang C, Yoon SA, Shin J (2020). Multifunctional sonosensitizers in sonodynamic cancer therapy. Chem Soc Rev.

[24] Nie Y, Chen W, Kang Y, Yuan X, Li Y, Zhou J (2023). Two-dimensional porous vermiculite-based nanocatalysts for synergetic catalytic therapy. Biomaterials.

[25] Chen H, Zhou X, Gao Y, Zheng B, Tang F, Huang J (2014). Recent progress in development of new sonosensitizers for sonodynamic cancer therapy. Drug Discov Today.

[26] Hu H, Zhao J, Ma K, Wang J, Wang X, Mao T (2023). Sonodynamic therapy combined with phototherapy: Novel synergistic strategy with superior efficacy for antitumor and antiinfection therapy. J Control Release.

[27] Zhang Y, Yang Y, Feng Y, Gao X, Pei L, Li X (2024). Sonodynamic therapy for the treatment of atherosclerosis. J Pharm Anal.

[28] Cressey P, Abd Shukor SB, Thanou M (2025). Sonodynamic therapy: transforming sound into light for hard-to-treat tumours. Adv Drug Deliv Rev.

[29] Zheng H, Yin N, Lv K, Niu R, Zhou S, Wang Y (2024). Defect-rich sonosensitizers based on CeO(2) with Schottky heterojunctions for boosting sonodynamic/chemodynamic synergistic therapy. J Mater Chem B.

[30] Cai J, Shen Q, Wu Y, Hu J, Pan D, Wu Y (2024). Defect Engineering of Biodegradable Sulfide Nanocage Sonozyme Systems Enables Robust Immunotherapy Against Metastatic Cancers. Adv Funct Mater.

[31] Chiba E, Ameur S, Ajjel R, Aloulou F, Altalhi T, Mezni A (2025). Enhanced Sonosensitizing and Dual-Enzyme Mimicking Activities of Au@TiO2-rGO Nanocomposite for Synergistic Medical Applications. BioNanoScience.

[32] Chiba E, Ameur S, Sammouda H, Altalhi T, Ajjel R, Mezni A (2025). Multifunctional Au@Pt-TiO2 Nanocomposite as Catalytic Cascade and Synergistic Sonodymaic Therapeutic Agents. J Inorg Organomet Polym Mater.

[33] Qin W, Cui J, Usama, Li Y, Liu R, Miao Z (2025). Lysosomal-Targeting and Light/Ultrasound Sensitive Carbon Dots for Combined Photodynamic and Sonodynamic Therapies of Breast Tumor. Adv Healthc Mater.

[34] Liu J, Xu Y, Ning H, Zhou Y, Chen C, Cong Z (2025). Folic acid-targeted Mn-doped carbon dots for controlled ROS amplification and FL/MR imaging-guided synergistic Sono-Chemodynamic Cancer therapy. J Colloid Interface Sci.

[35] Geng B, Hu J, Li Y, Feng S, Pan D, Feng L (2022). Near-infrared phosphorescent carbon dots for sonodynamic precision tumor therapy. Nat Commun.

[36] Chen Z, Sang L, Liu Y, Bai Z (2025). Sono-Piezo Dynamic Therapy: Utilizing Piezoelectric Materials as Sonosensitizer for Sonodynamic Therapy. Adv Sci (Weinh).

[37] Chen Z, Yang L, Yang Z, Wang Z, He W, Zhang W (2023). Disordered Convolution Region of P(VDF-TrFE) Piezoelectric Nanoparticles: The Core of Sono-Piezo Dynamic Therapy. ACS Appl Mater Interfaces.

[38] Chen Z, Yang L, Yang Z, Wang Z, He W, Zhang W (2024). Ultrasonic-responsive piezoelectric stimulation enhances sonodynamic therapy for HER2-positive breast cancer. J Nanobiotechnology.

[39] Chiba E, Ameur S, Sammouda H, Altalhi T, Ajjel R, Mezni A (2025). Multifunctional Au@ Pt-TiO2 Nanocomposite as Catalytic Cascade and Synergistic Sonodymaic Therapeutic Agents. J Inorg Organomet Polym Mater.

[40] Jiang S, Li H, Zhang L, Mu W, Zhang Y, Chen T (2025). Generic Diagramming Platform (GDP): a comprehensive database of high-quality biomedical graphics. Nucleic Acids Res.

[41] Zhu P, Chen Y, Shi J (2020). Piezocatalytic Tumor Therapy by Ultrasound-Triggered and BaTiO(3) -Mediated Piezoelectricity. Adv Mater.

[42] Chen Z, Sang L, Bian D, Liu Y, Bai Z (2025). Piezo-catalytic immunotherapy: mechanisms and feasibility in cancer treatment. Theranostics.

[43] Lu D, Wang L, Wang L, An L, Huo M, Xu H (2022). Probiotic Engineering and Targeted Sonoimmuno-Therapy Augmented by STING Agonist. Adv Sci (Weinh).

[44] Li D, Yang Y, Li D, Pan J, Chu C, Liu G (2021). Organic Sonosensitizers for Sonodynamic Therapy: From Small Molecules and Nanoparticles toward Clinical Development. Small.

[45] Dong HQ, Fu XF, Wang MY, Zhu J (2023). Research progress on reactive oxygen species production mechanisms in tumor sonodynamic therapy. World J Clin Cases.

[46] Matsufuji S, Kitajima Y, Higure K, Kimura N, Maeda S, Yamada K (2023). A HIF-1alpha inhibitor combined with palmitic acid and L-carnitine treatment can prevent the fat metabolic reprogramming under hypoxia and induce apoptosis in hepatocellular carcinoma cells. Cancer Metab.

[47] Liu W, Shao R, Guo L, Man J, Zhang C, Li L (2024). Precise Design of TiO(2)@CoO(x) Heterostructure via Atomic Layer Deposition for Synergistic Sono-Chemodynamic Oncotherapy. Adv Sci (Weinh).

[48] Liu Y, Huang Y, Lu P, Ma Y, Xiong L, Zhang X (2023). Manganese Dioxide/Gold-based Active Tumor Targeting Nanoprobes for Enhancing Photodynamic and Low-Temperature-Photothermal Combination Therapy in Lung Cancer. ACS Appl Mater Interfaces.

[49] Zhang Z, Zeng W, Guo N, Ran M, Gan H, Wu Q (2025). A nanodrug loading indocyanine green and metformin dually alleviating tumor hypoxia for enhanced chemodynamic/sonodynamic therapy. J Colloid Interface Sci.

[50] Zhang B, Zhang Y, Dang W, Xing B, Yu C, Guo P (2022). The anti-tumor and renoprotection study of E-[c(RGDfK)(2)]/folic acid co-modified nanostructured lipid carrier loaded with doxorubicin hydrochloride/salvianolic acid A. J Nanobiotechnology.

[51] Dong S, Liang S, Cheng Z, Zhang X, Luo L, Li L (2022). ROS/PI3K/Akt and Wnt/beta-catenin signalings activate HIF-1alpha-induced metabolic reprogramming to impart 5-fluorouracil resistance in colorectal cancer. J Exp Clin Cancer Res.

[52] Xie X, Zhang J, Sun L, Xu S, Ma SS, Wang H (2025). Ultrasound-triggered topical oxygen delivery enhances synergistic sonodynamic and antibody therapies against hypoxic gastric cancer. J Control Release.

[53] Indira Chandran V, Mansson AS, Barbachowska M, Cerezo-Magana M, Nodin B, Joshi B (2020). Hypoxia Attenuates Trastuzumab Uptake and Trastuzumab-Emtansine (T-DM1) Cytotoxicity through Redistribution of Phosphorylated Caveolin-1. Mol Cancer Res.

[54] Wang Y, Lv B, Wang H, Ren T, Jiang Q, Qu X (2024). Ultrasound-Triggered Azo Free Radicals for Cervical Cancer Immunotherapy. ACS Nano.

[55] Dunbar CE, High KA, Joung JK, Kohn DB, Ozawa K, Sadelain M (2018). Gene therapy comes of age. Science.

[56] Li Y, Liu Y, Xu J, Chen D, Wu T, Cao Y (2023). Macrophage-Cancer Hybrid Membrane-Camouflaged Nanoplatforms for HIF-1alpha Gene Silencing-Enhanced Sonodynamic Therapy of Glioblastoma. ACS Appl Mater Interfaces.

[57] Wang Y (2023). Bacterial therapy: a promising strategy for cancer immunotherapy. Cancer Biol Med.

[58] Taniguchi S, Fujimori M, Sasaki T, Tsutsui H, Shimatani Y, Seki K (2010). Targeting solid tumors with non-pathogenic obligate anaerobic bacteria. Cancer Sci.

[59] Wang T, Du M, Yuan Z, Guo J, Chen Z (2024). Multi-functional nanosonosensitizer-engineered bacteria to overcome tumor hypoxia for enhanced sonodynamic therapy. Acta Biomater.

[60] Manohar S, Gambhir SS (2020). Clinical photoacoustic imaging. Photoacoustics.

[61] Gao Y, Li Y, Cao H, Jia H, Wang D, Ren C (2022). Hypertoxic self-assembled peptide with dual functions of glutathione depletion and biosynthesis inhibition for selective tumor ferroptosis and pyroptosis. J Nanobiotechnology.

[62] Yang Y, Wang N, Wang Z, Yan F, Shi Z, Feng S (2025). Glutathione-Responsive Metal-Organic-Framework-Derived Mn(x)O(y)/(A/R)TiO(2) Nanoparticles for Enhanced Synergistic Sonodynamic/Chemodynamic/Immunotherapy. ACS Nano.

[63] Feng C, Wang L, Zhang D, Geng L, Zhou L, Wang L (2024). Tumour microenvironment-responded Fe-doped carbon dots-sensitized cubic Cu(2)O for Z-scheme heterojunction-enhanced sono-chemodynamic synergistic tumor therapy. J Colloid Interface Sci.

[64] Sun N, Lei Q, Wu M, Gao S, Yang Z, Lv X (2024). Metal-organic framework-mediated siRNA delivery and sonodynamic therapy for precisely triggering ferroptosis and augmenting ICD in osteosarcoma. Mater Today Bio.

[65] Xu P, Wen C, Gao C, Liu H, Li Y, Guo X (2024). Near-Infrared-II-Activatable Self-Assembled Manganese Porphyrin-Gold Heterostructures for Photoacoustic Imaging-Guided Sonodynamic-Augmented Photothermal/Photodynamic Therapy. ACS Nano.

[66] Wang C, Guo Y, Tan G, Kang W, Guo W, Song N (2025). Interfacial Engineering of Pillararene-Modified Ceria Nanoparticles for Regulable Enhanced Sonodynamic Therapy. Aggregate.

[67] Cao C, Wang X, Yang N, Song X, Dong X (2022). Recent advances of cancer chemodynamic therapy based on Fenton/Fenton-like chemistry. Chem Sci.

[68] Feng L, Gai S, He F, Yang P, Zhao Y (2020). Multifunctional Bismuth Ferrite Nanocatalysts with Optical and Magnetic Functions for Ultrasound-Enhanced Tumor Theranostics. ACS Nano.

[69] Jana D, Zhao Y (2022). Strategies for enhancing cancer chemodynamic therapy performance. Exploration (Beijing).

[70] Du MY, Xiao HP, Xu RF, Sun XB, Wang XY, Li DM (2025). A Sonosensitive Heterometallic Polyoxometalate for Highly Efficient Chemo-Sonodynamic Synergistic Cancer Therapy. Angew Chem Int Ed Engl.

[71] Zhao Y, Wang S, Ding Y, Zhang Z, Huang T, Zhang Y (2022). Piezotronic Effect-Augmented Cu(2-x)O-BaTiO(3) Sonosensitizers for Multifunctional Cancer Dynamic Therapy. ACS Nano.

[72] Fu S, Yang R, Ren J, Liu J, Zhang L, Xu Z (2021). Catalytically Active CoFe(2)O(4) Nanoflowers for Augmented Sonodynamic and Chemodynamic Combination Therapy with Elicitation of Robust Immune Response. ACS Nano.

[73] Kobayashi H, Choyke PL (2019). Near-Infrared Photoimmunotherapy of Cancer. Acc Chem Res.

[74] Geng P, Li Y, Macharia DK, Ren X, Meng R, Wang W (2024). One Stone, Three Birds: Design and Synthesis of "All-in-One" Nanoscale Mn-Porphyrin Coordination Polymers for Magnetic Resonance Imaging-Guided Synergistic Photodynamic-Sonodynamic Therapy. J Colloid Interface Sci.

[75] Xu D, Dai Q, Sun J, An Y, Wang Z, Yang L (2025). Optimizing energy transfer in NIR ruthenium(II) complexes to enhance stability and efficiency in sonodynamic therapy. Ultrason Sonochem.

[76] Karges J, Heinemann F, Jakubaszek M, Maschietto F, Subecz C, Dotou M (2020). Rationally Designed Long-Wavelength Absorbing Ru(II) Polypyridyl Complexes as Photosensitizers for Photodynamic Therapy. J Am Chem Soc.

[77] Roque Iii JA, Cole HD, Barrett PC, Lifshits LM, Hodges RO, Kim S (2022). Intraligand Excited States Turn a Ruthenium Oligothiophene Complex into a Light-Triggered Ubertoxin with Anticancer Effects in Extreme Hypoxia. J Am Chem Soc.

[78] Meng Z, Chao Y, Zhou X, Liang C, Liu J, Zhang R (2018). Near-Infrared-Triggered in Situ Gelation System for Repeatedly Enhanced Photothermal Brachytherapy with a Single Dose. ACS Nano.

[79] An J, He X, Ma H, Li Y, Li Y, Zhang X (2023). Radionuclide labeled nanocarrier for imaging guided combined radionuclide, sonodynamic, and photothermal therapy of pancreatic tumours. J Colloid Interface Sci.

[80] Frangioni JV (2003). In vivo near-infrared fluorescence imaging. Curr Opin Chem Biol.

[81] Dagher OK, Schwab RD, Brookens SK, Posey AD Jr (2023). Advances in cancer immunotherapies. Cell.

[82] Li J, Luo Y, Zeng Z, Cui D, Huang J, Xu C (2022). Precision cancer sono-immunotherapy using deep-tissue activatable semiconducting polymer immunomodulatory nanoparticles. Nat Commun.

[83] Gupta R, Mehta A, Wajapeyee N (2022). Transcriptional determinants of cancer immunotherapy response and resistance. Trends Cancer.

[84] Zhang Q, Bao C, Cai X, Jin L, Sun L, Lang Y (2018). Sonodynamic therapy-assisted immunotherapy: A novel modality for cancer treatment. Cancer Sci.

[85] Ding R, Yang H, Wang J, Liu Y, Mu S, Wang D (2025). Advances in Stimuli-Responsive Release Strategies for Sonosensitizers in Synergistic Sonodynamic Immunotherapy against Tumors. Adv Healthc Mater.

[86] Chen Q, Xiao H, Hu L, Huang Y, Cao Z, Shuai X (2024). (19)F MRI/CEUS Dual Imaging-Guided Sonodynamic Therapy Enhances Immune Checkpoint Blockade in Triple-Negative Breast Cancer. Adv Sci (Weinh).

[87] Fang J, Xu R, Cao Y, Zhao Z, Li W, Lin L (2025). Reduction-responsive RNAi nanoplatform for enhanced cancer sonoimmunotherapy via dual inhibition of mitophagy and Nrf2 pathways. Theranostics.

[88] Yang H, Li R, Jin S, Tian Y, Wang C, Sun Y (2025). Targeted nanosensitizer-augmented sono-immunotherapy with STING agonist to remodel the immune microenvironment in hepatocellular carcinoma. Acta Biomater.

[89] Pu H, Huang J, Gui B, Chen Y, Guo Y, Lian Y (2025). Ultrasound-Responsive Nanobubbles for Breast Cancer: Synergistic Sonodynamic, Chemotherapy, and Immune Activation through the cGAS-STING Pathway. ACS Appl Mater Interfaces.

[90] Zeng X, Wang X, Zhao Y, Guo L, Sun X, Shang M (2025). Dual-mode immunotherapy: ultrasound responsive zinc-based nano-contract agents synergistically activate the GAS-STING pathway for enhanced tumor sono-metalloimmunotherapy. Ultrason Sonochem.

[91] Huang H, Du L, Su R, Li Z, Shao Y, Yuan Y (2024). Albumin-based co-loaded sonosensitizer and STING agonist nanodelivery system for enhanced sonodynamic and immune combination antitumor therapy. J Control Release.

[92] Geng B, Hu J, He X, Zhang Z, Cai J, Pan D (2024). Single Atom Catalysts Remodel Tumor Microenvironment for Augmented Sonodynamic Immunotherapy. Adv Mater.

[93] Ghosh C, Luong G, Sun Y (2021). A snapshot of the PD-1/PD-L1 pathway. J Cancer.

[94] Thomas-Jardin S, Suresh S, Arce A, Novaresi N, Deng Q, Stein E (2025). The Integrated Stress Response Pathway Coordinates Translational Control of Multiple Immune Checkpoints in Lung Cancer. Cancer Res.

[95] Dutta B, Shelar SB, Nirmalraj A, Gupta S, Barick KC, Gupta J (2023). Smart Magnetic Nanocarriers for Codelivery of Nitric Oxide and Doxorubicin for Enhanced Apoptosis in Cancer Cells. ACS Omega.

[96] Luo Y, Qi X, Zhang Z, Zhang J, Li B, Shu T (2024). Inactivation of Malic Enzyme 1 in Endothelial Cells Alleviates Pulmonary Hypertension. Circulation.

[97] Mazuryk O, Gurgul I, Oszajca M, Polaczek J, Kieca K, Bieszczad-Zak E (2024). Nitric Oxide Signaling and Sensing in Age-Related Diseases. Antioxidants (Basel).

[98] Song Y, Xu S, Zhang J, Zhang T, Wu R, Feng G (2025). Ultrasound-Mediated Piezocatalysis Triggers NO Release to Augment Targeted Immunotherapy of Pancreatic Cancer. ACS Nano.

[99] Wang P, Tang Q, Zhang L, Xu M, Sun L, Sun S (2021). Ultrasmall Barium Titanate Nanoparticles for Highly Efficient Hypoxic Tumor Therapy via Ultrasound Triggered Piezocatalysis and Water Splitting. ACS Nano.

[100] Wang Z, Zhang F, Zhou B, Sun L, Liu B, Liu M (2025). Gradient-driven deep penetration of self-electrophoretic nanoparticles in acidic tumor microenvironments for enhanced antitumor therapy. Biomaterials.

[101] Lotscher J, Marti ILAA, Kirchhammer N, Cribioli E, Giordano Attianese GMP, Trefny MP (2022). Magnesium sensing via LFA-1 regulates CD8(+) T cell effector function. Cell.

[102] Boroughs LK, DeBerardinis RJ (2015). Metabolic pathways promoting cancer cell survival and growth. Nat Cell Biol.

[103] Hurley HJ, Dewald H, Rothkopf ZS, Singh S, Jenkins F, Deb P (2021). Frontline Science: AMPK regulates metabolic reprogramming necessary for interferon production in human plasmacytoid dendritic cells. J Leukoc Biol.

[104] Guerra L, Bonetti L, Brenner D (2020). Metabolic Modulation of Immunity: A New Concept in Cancer Immunotherapy. Cell Rep.

[105] Peng H, Wang D, Huang S, Yu A (2025). Dual-targeting Aggregation-induced emission polymer micelles mediate immunogenic sonodynamic therapy for Tumor cell growth inhibition and macrophage reprogramming. Acta Biomater.

[106] Yu L, Gao L, Liang B, Zhang L, Wu M, Liu J (2025). Polymer-based nanodrugs enhance sonodynamic therapy through epigenetic reprogramming of the immunosuppressive tumor microenvironment. J Control Release.

[107] Zheng J, Zhao F, Pariente E, Xu X, Zhang X, Shabiti S (2025). Tumor-Targeted Glutamine Metabolism Blocker Synergizes with TiO(2)-Au Janus Nanoparticles for Enhanced Sono-Metabolic Antitumor Therapy. Adv Mater.

[108] Aymeric L, Apetoh L, Ghiringhelli F, Tesniere A, Martins I, Kroemer G (2010). Tumor cell death and ATP release prime dendritic cells and efficient anticancer immunity. Cancer Res.

[109] Elliott MR, Chekeni FB, Trampont PC, Lazarowski ER, Kadl A, Walk SF (2009). Nucleotides released by apoptotic cells act as a find-me signal to promote phagocytic clearance. Nature.

[110] Uhlen M, Fagerberg L, Hallstrom BM, Lindskog C, Oksvold P, Mardinoglu A (2015). Proteomics. Tissue-based map of the human proteome. Science.

[111] Huang S, Apasov S, Koshiba M, Sitkovsky M (1997). Role of A2a extracellular adenosine receptor-mediated signaling in adenosine-mediated inhibition of T-cell activation and expansion. Blood.

[112] Timperi E, Barnaba V (2021). CD39 Regulation and Functions in T Cells. Int J Mol Sci.

[113] Zhang Y, Jin W, Deng Z, Gao B, Zhu Y, Fu J (2025). Metabolic reprogramming nanomedicine potentiates colon cancer sonodynamic immunotherapy by inhibiting the CD39/CD73/ADO pathway. Acta Pharm Sin B.

[114] Bacci M, Lorito N, Smiriglia A, Morandi A (2021). Fat and Furious: Lipid Metabolism in Antitumoral Therapy Response and Resistance. Trends Cancer.

[115] Duan Y, Deng M, Liu B, Meng X, Liao J, Qiu Y (2024). Mitochondria targeted drug delivery system overcoming drug resistance in intrahepatic cholangiocarcinoma by reprogramming lipid metabolism. Biomaterials.

[116] Wang Y, Wu J, Chen M, Zhang J, Sun X, Zhou H (2024). Application of near-infrared-activated and ATP-responsive trifunctional upconversion nano-jelly for in vivo tumor imaging and synergistic therapy. Biosens Bioelectron.

[117] Wang L, Liu Y, Sun J, Su J, Feng J, Miao L (2025). In Situ Ultrasound-Triggered Bioluminescence for Combined Sono/Photodynamic Immunotherapy. ACS Nano.

[118] Xu C, Dong J, Shi X, Rui J, Chen M, Lu W (2025). Engineered microalgae for photo-sonodynamic synergistic therapy in breast cancer treatment. Acta Biomater.

[119] Gao C, Kwong CHT, Wang Q, Kam H, Xie B, Lee SM (2023). Conjugation of Macrophage-Mimetic Microalgae and Liposome for Antitumor Sonodynamic Immunotherapy via Hypoxia Alleviation and Autophagy Inhibition. ACS Nano.

[120] Smalley JW, Olczak T (2017). Heme acquisition mechanisms of Porphyromonas gingivalis - strategies used in a polymicrobial community in a heme-limited host environment. Mol Oral Microbiol.

[121] Yang M, Meng Y, Li J, Bai L, Cheng Y, Liu Y (2025). Nanofabricated Bacterial Cell Walls with Intrinsic Peroxidase-Mimicking and Sonodynamic Activities for Cancer Combination Treatment. Adv Sci (Weinh).

[122] Gong Z, Mao Y, Liu Y, Hu X, Zhang Y, Zhu L (2024). Sono-promoted piezocatalysis and low-dose drug penetration for personalized therapy via tumor organoids. J Colloid Interface Sci.

[123] Li HT, Zhao J, Lu JY, Peng XC, Han N, Li LG (2025). Cepharanthine Loaded TCPP-MOF Triggers Pyroptosis Through TSPO Inhibition for Hepatocellular Carcinoma Sonodynamic-Chemotherapy. Adv Healthc Mater.

[124] Shi Z, Zeng Y, Luo J, Wang X, Ma G, Zhang T (2024). Endogenous Magnetic Lipid Droplet-Mediated Cascade-Targeted Sonodynamic Therapy as an Approach to Reversing Breast Cancer Multidrug Resistance. ACS Nano.

[125] Su Z, Dong S, Zhao SC, Liu K, Tan Y, Jiang X (2021). Novel nanomedicines to overcome cancer multidrug resistance. Drug Resist Updat.

[126] Halaby R (2019). Influence of lysosomal sequestration on multidrug resistance in cancer cells. Cancer Drug Resist.

[127] Mlejnek P, Havlasek J, Pastvova N, Dolezel P, Dostalova K (2022). Lysosomal sequestration of weak base drugs, lysosomal biogenesis, and cell cycle alteration. Biomed Pharmacother.

[128] Nie T, Zou W, Meng Z, Wang L, Ying T, Cai X (2022). Bioactive Iridium Nanoclusters with Glutathione Depletion Ability for Enhanced Sonodynamic-Triggered Ferroptosis-Like Cancer Cell Death. Adv Mater.

[129] Yang X, Wang L, Guo S, Li R, Tian F, Guan S (2021). Self-Cycling Free Radical Generator from LDH-Based Nanohybrids for Ferroptosis-Enhanced Chemodynamic Therapy. Adv Healthc Mater.

[130] Meng W, Chen T, Li X, Li Y, Zhang L, Xu Y (2025). A Dual-Targeting Biomimetic Nanoplatform Integrates SDT/CDT/Gas Therapy to Boost Synergistic Ferroptosis for Orthotopic Hepatocellular Carcinoma Therapy. Adv Sci (Weinh).

[131] Chen J, Zhan Q, Li L, Xi S, Cai L, Liu R (2025). Cell-membrane targeting sonodynamic therapy combination with FSP1 inhibition for ferroptosis-boosted immunotherapy. Mater Today Bio.

[132] Shi Y, Fan G, Yang E, Zhang Y, Ding H, Tian J (2025). Enhanced efficacy of immune checkpoint inhibitors by folate-targeted multifunctional drug through synergistic therapy inducing ferroptosis and immunogenic cell death in bladder cancer. Mater Today Bio.

[133] Wu M, Zhang Z, Li D, Ruan X, Yang J, Chen S (2025). Integrating oxygen-boosted sonodynamic therapy and ferroptosis via engineered exosomes for effective cancer treatment. Theranostics.

[134] Zhao J, Bian E, Zhang R, Xu T, Nie Y, Wang L (2024). Self-Assembled Aza-Boron-Dipyrromethene-Based H(2)S Prodrug for Synergistic Ferroptosis-Enabled Gas and Sonodynamic Tumor Therapies. Adv Sci (Weinh).

[135] Li SR, Bu LL, Cai L (2022). Cuproptosis: lipoylated TCA cycle proteins-mediated novel cell death pathway. Signal Transduct Target Ther.

[136] Cui PP, Yang QC, Sun ZJ (2026). Targeting cuproptosis with nanomaterials for cancer immunotherapy. Acta Biomater.

[137] Heras-Murillo I, Adan-Barrientos I, Galan M, Wculek SK, Sancho D (2024). Dendritic cells as orchestrators of anticancer immunity and immunotherapy. Nat Rev Clin Oncol.

[138] Li Y, Liu J, Weichselbaum RR, Lin W (2024). Mitochondria-Targeted Multifunctional Nanoparticles Combine Cuproptosis and Programmed Cell Death-1 Downregulation for Cancer Immunotherapy. Adv Sci (Weinh).

[139] Hu H, Yan J, Zhu H, Wang X, Zhao Y, Li S (2024). Self-delivered sonodynamic nanomedicine for enhanced tumor immunotherapy by simultaneously reversing the immunosuppression and immune resistance. Chem Eng J.

[140] Hu J, Yan L, Cao Z, Geng B, Cao X, Liu B (2024). Tumor Microenvironment Activated Cu Crosslinked Near-Infrared Sonosensitizers for Visualized Cuproptosis-Enhanced Sonodynamic Cancer Immunotherapy. Adv Sci (Weinh).

[141] Yan L, Chang L, Tian Y, Hu J, Cao Z, Guo X (2025). Graphene Quantum Dot Sensitized Heterojunctions Induce Tumor-Specific Cuproptosis to Boost Sonodynamic and Chemodynamic Enhanced Cancer Immunotherapy. Adv Sci (Weinh).

[142] Tang C, Liu K, Gao X, Kang H, Xie W, Chang J (2025). A metal-organic framework functionalized CaO(2)-based cascade nanoreactor induces synergistic cuproptosis/ferroptosis and Ca(2+) overload-mediated mitochondrial damage for enhanced sono-chemodynamic immunotherapy. Acta Biomater.

[143] Zhong X, Li X, Gu L, Yang H, Du J, Wang Q (2025). Piezoelectric-mediated two-dimensional copper-based metal-organic framework for synergistic sonodynamic and cuproptosis-driven tumor therapy. J Colloid Interface Sci.

[144] Tulinska J, Mikusova ML, Liskova A, Busova M, Masanova V, Uhnakova I (2022). Copper Oxide Nanoparticles Stimulate the Immune Response and Decrease Antioxidant Defense in Mice After Six-Week Inhalation. Front Immunol.

[145] Falcone E, Ritacca AG, Hager S, Schueffl H, Vileno B, El Khoury Y (2022). Copper-Catalyzed Glutathione Oxidation is Accelerated by the Anticancer Thiosemicarbazone Dp44mT and Further Boosted at Lower pH. J Am Chem Soc.

[146] Cao X, Mao L, Tian Y, Yan L, Geng B, Zhou Y (2025). In situ construction of heterojunctions to regulate the biodegradation behavior of copper carriers for tumor-specific cuproptosis-enhanced sono-immunotherapy. J Nanobiotechnology.

[147] Xu Y, Liu SY, Zeng L, Ma H, Zhang Y, Yang H (2022). An Enzyme-Engineered Nonporous Copper(I) Coordination Polymer Nanoplatform for Cuproptosis-Based Synergistic Cancer Therapy. Adv Mater.

[148] Wang Y, Yan T, Cai J, Dou H, Zhu Y, Geng B (2025). A heterojunction-engineering nanodrug with tumor microenvironment responsiveness for tumor-specific cuproptosis and chemotherapy amplified sono-immunotherapy. Biomaterials.

[149] Allen C, Her S, Jaffray DA (2017). Radiotherapy for Cancer: Present and Future. Adv Drug Deliv Rev.

[150] Citrin DE (2017). Recent Developments in Radiotherapy. N Engl J Med.

[151] Li X, Sun Y, Wang Y, Zhou Y, Bao Y, Zhang Z (2025). Amplifying Radiotherapy by Evoking Mitochondrial Oxidative Stress using a High-performance Aggregation-induced Emission Sonosensitizer. Curr Med Chem.

[152] Jiang Y, Liao X, Tang W, Huang C, Pan Y, Ning S (2024). Platelet Membrane Biomimetic Manganese Carbonate Nanoparticles Promote Breast Cancer Stem Cell Clearance for Sensitized Radiotherapy. Int J Nanomedicine.

[153] Liu J, Shi M, Zhao H, Bai X, Lin Q, Guan X (2025). Ultrasound-activated nano-oxygen sensitizer for sonodynamic-radiotherapy of esophageal cancer. Nanoscale Adv.

[154] Huangfu L, Zha B, Li P, Wang L, Liu X, Cui H (2025). A phase I clinical trial of sonodynamic therapy combined with radiotherapy for brainstem gliomas. Int J Cancer.

[155] Zhang Y, Wang Y, Zhu A, Yu N, Xia J, Li J (2024). Dual-Targeting Biomimetic Semiconducting Polymer Nanocomposites for Amplified Theranostics of Bone Metastasis. Angew Chem Int Ed Engl.

[156] Zhu M, Liu J, Li Y, Ya Z, Liang M, Zhang L (2026). A controllable self-amplifying oxidative stress strategy for boosting noninvasive sonodynamic therapy and synergistic immunotherapy. Biomaterials.

[157] Zhou S, Gravekamp C, Bermudes D, Liu K (2018). Tumour-targeting bacteria engineered to fight cancer. Nat Rev Cancer.

[158] Wu MR, Jusiak B, Lu TK (2019). Engineering advanced cancer therapies with synthetic biology. Nat Rev Cancer.

[159] Yang G, Phua SZF, Bindra AK, Zhao Y (2019). Degradability and Clearance of Inorganic Nanoparticles for Biomedical Applications. Adv Mater.

[160] Canale FP, Basso C, Antonini G, Perotti M, Li N, Sokolovska A (2021). Metabolic modulation of tumours with engineered bacteria for immunotherapy. Nature.

[161] Yang Z, Jiao Z, Chen Z, Qiao C, Huang C, Wang L (2025). Programmable Bacterial Architects Crafting Sonosensitizers for Tumor-Specific Sonodynamic Immunotherapy. Adv Mater.

[162] Ho CL, Tan HQ, Chua KJ, Kang A, Lim KH, Ling KL (2018). Engineered commensal microbes for diet-mediated colorectal-cancer chemoprevention. Nat Biomed Eng.

[163] Pan P, Dong X, Chen Y, Zeng X, Zhang XZ (2022). Engineered Bacteria for Enhanced Radiotherapy against Breast Carcinoma. ACS Nano.

[164] Ma X, Liang X, Li Y, Feng Q, Cheng K, Ma N (2023). Modular-designed engineered bacteria for precision tumor immunotherapy via spatiotemporal manipulation by magnetic field. Nat Commun.

[165] Wang K, Xiang Y, Pan W, Wang H, Li N, Tang B (2020). Dual-targeted photothermal agents for enhanced cancer therapy. Chem Sci.

[166] Gao Y, Ouyang Z, Shen S, Yu H, Jia B, Wang H (2023). Manganese Dioxide-Entrapping Dendrimers Co-Deliver Protein and Nucleotide for Magnetic Resonance Imaging-Guided Chemodynamic/Starvation/Immune Therapy of Tumors. ACS Nano.

[167] Ying W, Zhang Y, Gao W, Cai X, Wang G, Wu X (2020). Hollow Magnetic Nanocatalysts Drive Starvation-Chemodynamic-Hyperthermia Synergistic Therapy for Tumor. ACS Nano.

[168] Yu J, He X, Wang Z, Liu S, Hao D, Li X (2021). Combination of starvation therapy and Pt-NP based chemotherapy for synergistic cancer treatment. J Mater Chem B.

[169] Wang X, Li Y, Jia F, Cui X, Pan Z, Wu Y (2022). Boosting nutrient starvation-dominated cancer therapy through curcumin-augmented mitochondrial Ca(2+) overload and obatoclax-mediated autophagy inhibition as supported by a novel nano-modulator GO-Alg@CaP/CO. J Nanobiotechnology.

[170] Xu S, Zhang H, Qian Z, Yuan W (2024). pH-Responsive injectable self-healing hydrogels loading Au nanoparticles-decorated bimetallic organic frameworks for synergistic sonodynamic-chemodynamic-starvation-chemo therapy of cancer. J Colloid Interface Sci.

[171] Cheng Y, Liu Q, Wang Y, Liu M, Mo Q, Zeng F (2025). Engineering hypoxia-specific core-shell nanotherapeutics: A sequential strategy for amplified multimodal synergistic breast cancer treatment. J Colloid Interface Sci.

[172] Wen M, Chen H, Xu S, Yang S, Guan X, Wang X (2025). Novel drug-free cascaded nanoparticles induce tumor-specific ROS storms via multimodal synergistic anticancer therapy. J Nanobiotechnology.

[173] Schaaf MB, Garg AD, Agostinis P (2018). Defining the role of the tumor vasculature in antitumor immunity and immunotherapy. Cell Death Dis.

[174] Jimenez-Jimenez C, Moreno VM, Vallet-Regi M (2022). Bacteria-Assisted Transport of Nanomaterials to Improve Drug Delivery in Cancer Therapy. Nanomaterials (Basel).

[175] Ma J, Chen CS, Blute T, Waxman DJ (2011). Antiangiogenesis enhances intratumoral drug retention. Cancer Res.

[176] Wang X, Zhong X, Bai L, Xu J, Gong F, Dong Z (2020). Ultrafine Titanium Monoxide (TiO(1+x)) Nanorods for Enhanced Sonodynamic Therapy. J Am Chem Soc.

[177] Chen Z, Sang L, Qixi Z, Li X, Liu Y, Bai Z (2025). Ultrasound-responsive nanoparticles for imaging and therapy of brain tumors. Mater Today Bio.

[178] Chen S, Zhu P, Mao L, Wu W, Lin H, Xu D (2023). Piezocatalytic Medicine: An Emerging Frontier using Piezoelectric Materials for Biomedical Applications. Adv Mater.

[179] Jiao X, Sun L, Zhang W, Ren J, Zhang L, Cao Y (2021). Engineering oxygen-deficient ZrO(2-x) nanoplatform as therapy-activated "immunogenic cell death (ICD)" inducer to synergize photothermal-augmented sonodynamic tumor elimination in NIR-II biological window. Biomaterials.

[180] Zhou Z, Wang T, Hu T, Xu H, Cui L, Xue B (2024). Synergistic Interaction between Metal Single-Atoms and Defective WO(3-) (x) Nanosheets for Enhanced Sonodynamic Cancer Therapy. Adv Mater.

[181] Lyu Y, Li Q, Xie S, Zhao Z, Ma L, Wu Z (2025). Synergistic Ultrasound-Activable Artificial Enzyme and Precision Gene Therapy to Suppress Redox Homeostasis and Malignant Phenotypes for Controllably Combating Hepatocellular Carcinoma. J Am Chem Soc.

